# Synthesis, Structure and Antioxidant Activity of Cyclohexene-Fused Selenuranes and Related Derivatives

**DOI:** 10.3390/molecules200712670

**Published:** 2015-07-13

**Authors:** Poonam Rajesh Prasad, Harkesh B. Singh, Ray J. Butcher

**Affiliations:** 1 Department of Chemistry, Indian Institute of Technology Bombay, Powai, Mumbai 400076, India; E-Mail: poonam@chem.iitb.ac.in; 2 Department of Chemistry, Howard University, Washington, DC 20059, USA; E-Mail: rbutcher99@yahoo.com

**Keywords:** spirodioxychalcogenuranes, glutathione peroxidase (GPx), antioxidant, monochalcogenides, Se–O linkage

## Abstract

Synthesis, structure and antioxidant activity of new cyclohexene-fused spiroselenuranes and a spirotellurane is reported. Oxidation reactions of bis(*o*-formylcyclohex-1-ene)selenide/bis(2-hydroxymethylcyclohex-1-ene)selenide provide the corresponding spiroselenuranes. The glutathione peroxidase-like activity of the newly synthesized compounds has been evaluated.

## 1. Introduction

**Scheme 1:**
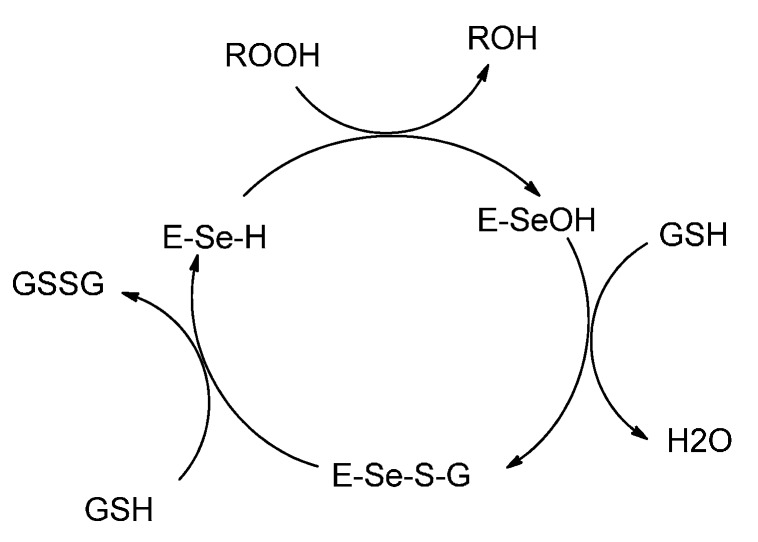
Catalytic mechanism of GPx enzyme.

The biochemistry of selenium is of considerable current interest due to the discovery of selenocysteine in a number of enzymes. The enzymes include glutathione peroxidase [1,2], iodothyronine deiodinase [3,4,5], and thioredoxin reductase *etc.* [6,7]. Glutathione peroxidase (GPx) functions as an antioxidant and is responsible for the destruction of harmful peroxides in various living organisms. The enzyme’s catalytic cycle involves selenol (Enz–SeH) as the active form that reduces hydroperoxides and becomes oxidized to the selenenic acid (ESeOH), which reacts with reduced glutathione (GSH) to form selenenyl sulfide adduct (ESeSG). A second molecule of glutathione then regenerates the active sites of the enzyme by attacking the selenosulfide (ESeSG) to form the oxidized glutathione (GSSG). Thus, in the overall catalytic cycle, two equivalents of glutathione are oxidized to the disulfide and water, while the hydroperoxide is reduced to the corresponding alcohol (Scheme 1) [8,9].

Recently, a series of organoselenium and -tellurium compounds which exhibit promising GPx-like antioxidant activity have been reported in the literature [10,11,12,13,14,15,16]. The compounds have either Se···O/N intramolecular interaction or Se–O/N linkage. The weak intramolecular interactions (Se···N/O) play an important role in stabilizing unstable organoselenium/-tellurium compounds and modulating the GPx-like activity of enzyme mimetics [17,18,19]. The important mimetics with Se–O/Se···O include; selenide **1** [20], cyclic seleninate ester **2** [21], diselenide **3** with intramolecular interaction [22], spirodioxychalcogenuranes **4**–**5** [23] and **6**–**9** and related compounds with Se–O linkage [24,25,26,27].

**Figure 1:**
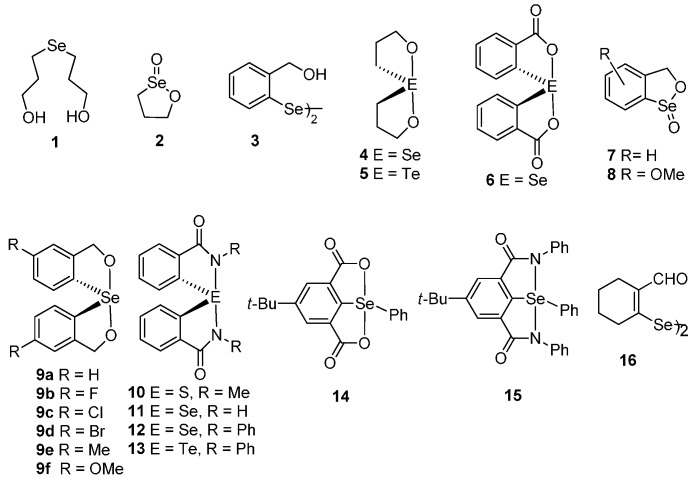
Some GPx mimics.

Back and coworkers reported the synthesis of a series of substituted aromatic spirocycles. The methoxy-substituted selenurane (**9f**) proved the most effective catalyst for the reduction of hydrogen peroxide with benzyl thiol. Detailed GPx-like activity of related spirodiazaselenurane/ellurane (**10**–**13)** have been evaluated by Back and coworkers [28] and Mugesh and coworkers [29,30]. Very recently, our group reported the synthesis and structure of bicyclic diacycloxy- and diazaselenuranes **14**–**15** and related compounds (Figure 1) [31]. Although there are many reports on the synthesis, structural studies and GPx-like activities of aromatic spirodioxychalcogenuranes and spirodiazaselenurane/-tellurane, studies on the aliphatic analogues especially alicyclic are rare. In this paper, we report the synthesis, structural characterization of some new alicyclic spirodioxyselenuranes and a spirodioxytellurane.

## 2. Results and Discussion

### 2.1. Synthesis

**Scheme 2:**
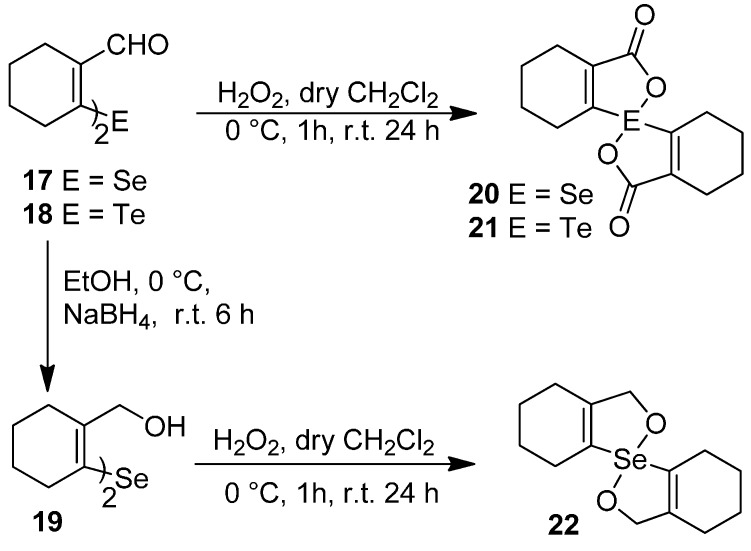
Synthesis of selenide **19** and spirodioxychalcogenuranes (**20**–**22**).

The precursors, di-(*o*-formylcyclohex-1-ene)diselenide **16** (Figure 1) and di-(*o*-formylcyclohex-1-ene)selenide **17** were obtained by the disodium diselenide route [32]. Di-(*o*-formylcyclohex-1-ene)telluride **18** was prepared by following the literature procedure [33]. Di(2-hydroxymethylcyclohex-1-ene)selenide **19** was obtained by the reduction of di-(*o*-formylcyclohex-1-ene)selenide **17** with NaBH_4_. Synthesis of spirodioxychalcogenuranes **20**–**22** was accomplished by the oxidation of monochalcogenides **17**–**19** with an excess of H_2_O_2_ (Scheme 2).

### 2.2. Spectroscopy Studies

The compounds were characterized by common spectroscopic tools such as IR, ^1^H-, ^77^Se-NMR spectroscopy, mass spectrometry and single crystal X-ray diffraction studies (Supplementary Materials). The FT-IR spectrum of **19** exhibits the OH stretching frequency (C–OH) at 3298 cm^−1^, indicating the presence of the alcohol group. The carbonyl stretching frequency of spirodioxyselenurane **20** and -tellurane **21** were observed at (ν_C=O_) 1683 cm^−1^ 1663 cm^−1^ respectively which are in good agreement with those reported for spirodioxyselenurane **6** (1695 cm^−1^) and 1,1′-spirobis(3*H*-2,1-benzoxatellurole)-3,3′-dione [34]. For compounds **19**–**21** the absence of the characteristic peak for the formyl group in ^1^H-NMR spectra confirmed the reduction or oxidation of **17**. This was further corroborated by single crystal X-ray analysis (*vide infra*). In the ^1^H-NMR spectrum of compound **22**, the methylene protons (–CH_2_–OH) are split. This is in contrast to the precursor **19**, where these appeared as a singlet. It is due to the diastereotopic nature of the methylene protons. A similar pattern has been reported by Back and coworkers for **9a** [35]. The ^77^Se-NMR spectrum of compound **22** exhibits a signal at 908 ppm. This is significantly downfield as compared to **19** (325 ppm).

### 2.3. X-ray Crystallographic Studies

#### 2.3.1. Molecular Structure of **19**

**Figure 2:**
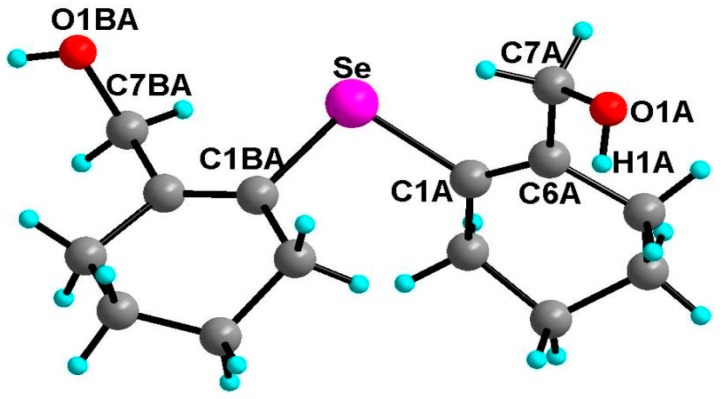
Molecular structure of **19**. Selected bond lengths (Å) and bond angles (°); Se–C7BA–H7BA···Se 2.710, C7BA–H7BA···Se 110.8, C7A–H7AB···Se 2.704, C7A–H7AB···Se 112.4, C1BA 1.935(5), Se–C1A 1.939(5), C1BA–Se–C1A 99.1(2), O1BA–C7BA–C6BA 111.8(4), O1A–C7A–C6A 114.2(4).

**Figure 3:**
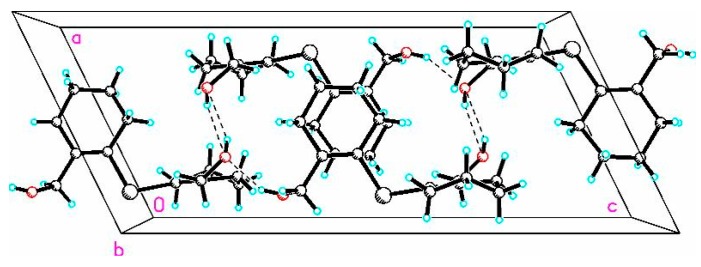
The short OH···O intermolecular interactions (OH···O, 2.163 Å, C7A–O1A···H1A 106.3° and OH···O, 2.040 Å, C7A–O1A···H1BA 103.1°).

The molecular structure of **19** is shown in Figure 2. The coordination geometry around the selenium atom is V**-**shaped with C1BA–Se–C1A bond angle being 99.10(1)°. A distinct feature of the structure is that both the hydroxyl groups are directed away from the selenium atom and there is asymmetry in the Se···OH bond distances. The Se···OH bond distances are 3.778 Å (Se–O1BA) and 4.486 Å (Se–O1A), respectively. A similar observation has been made in the case of the bis(*o*-formylphenyl) selenide having two formyl groups *trans* to the selenium [36]. Selvakumar *et al.* also found a similar situation in the case of a (5-(*tert*-butyl)-2-(phenylselanyl)-1,3-phenylene)dimethanol having two hydroxyl groups where none were coordinated to the selenium [31]. However, in di(2-hydroxybenzyl)selenide [22], out of the two OH groups, one OH group was coordinated to selenium with a bond distance of 3.008 Å. The lack of coordinating ability of the CH_2_OH group in **19** as well as in its aromatic analogue may be due to the poor electron donating property of the CH_2_OH groups and the availability of rotational degrees of freedom which could prevent the O···Se···O repulsive interaction. The compound shows hydrogen bonding interactions between the hydroxyl groups of the adjacent molecules (Figure 3).

Interestingly, in case of **19**, along with Se···OH intermolecular interaction, short C–H···Se intramolecular interactions were also observed. The short interatomic distances between selenium and one of the benzylic hydrogens (C–H···Se) are 2.710 and 2.704 Å which are shorter than the reported for diselenocin [37] (2.92 Å) and sum of the van der Walls radii (2.99 Å) [38]. The C–H···Se bond angles for **19** are 110.8° and 112.4° which are good agreement with diselenocin (101.7° and 107.0°). The solid state IR (KBr) spectrum indicates Se···H–C interaction. The C–H symmetric stretching frequency (2831 cm^−1^) is shifted towards the lower wave number as compared with normal methylene adjacent to the electronegative atom (υ = 2855 cm^−1^).

**Figure 4:**
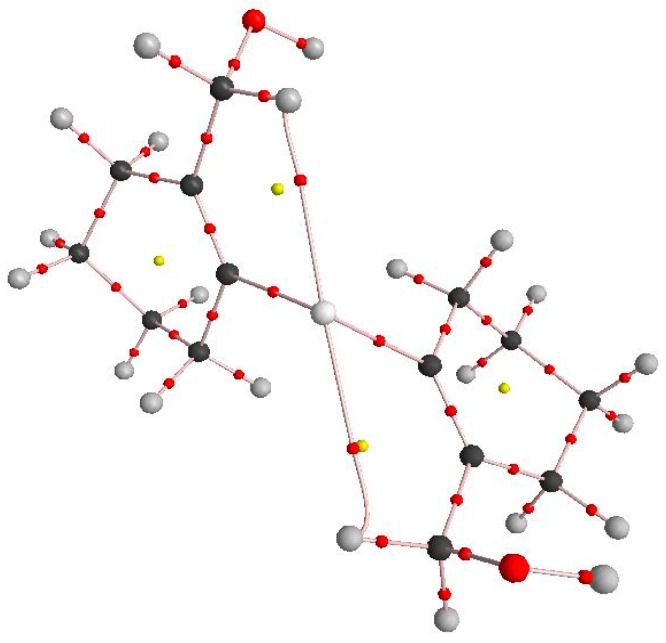
AIM picture of **19** showing bond critical point for C–H···Se interaction.

To gain more information about the intramolecular Se···H interaction, density functional theory calculations have been carried out using Gaussian09 suite of programs [39]. The geometry of **19** was optimized at B3LYP/6–31+g(d) basis set. The Natural Bond Orbital (NBO) second-order perturbation energies for the C–H···Se interaction for **19** are *E*_C-H···Se_ 2.49 and 1.40 kcal mol^−1^. It was further confirmed by Atoms in Molecules (AIM) analysis. The AIM [40,41,42] analysis was performed with AIM [43] at B3LYP/6-31+g(d) level of theory with wtbs basis set for Se and 6–31+g(d) level for remaining atoms. On carrying out AIM analysis of **19**, the bond critical point was located in between C–H–Se which confirms the presence of C–H···Se intramolecular interaction (Figure 4). The values of electron density for C–H···Se were (ρ) 0.0124 and 0.0123 a.u.

#### 2.3.2. Molecular Structure of **20**

**Figure 5:**
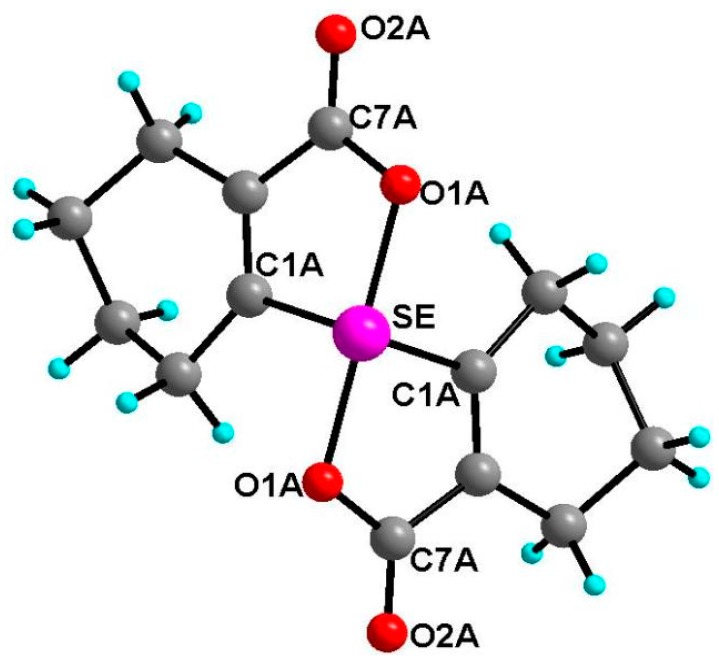
Molecular structure of compound **20**. Selected bond lengths (Å) and bond angles (°); Se–C1A 1.939(2), Se–O1A 1.970(1), Se–O1A 1.970(1), C7A–O2A 1.213(1), C1A–Se–C1A 99.27(9), O1A–Se–O1A 172.99(7).

**Figure 6:**
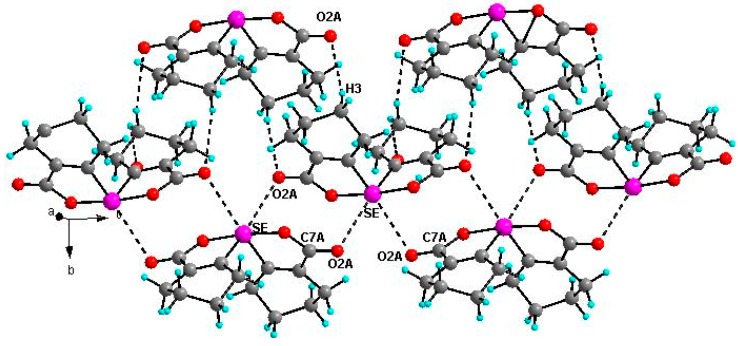
Two-dimensional packing diagram of compound **20** O2A···Se 3.053(2) Å, C7A–O2A–Se 102.8(2)°, O2A···H3 2.609(2) Å, C3A–H3–O2A 157.7(3)°.

Dioxyselenurane **20** crystallizes in a monoclinic crystal system with *C*2/c space group (Figure 5). The geometry around the central atom is distorted trigonal bipyramidal in which two Se–O bonds occupy the axial positions, two Se–C bonds and the lone pair occupy the equatorial positions. The Se–O bond length of 1.971(1) Å is slightly elongated compared to the covalent Se–O bond length (1.83 Å) and in good agreement with reported Se–O bond distance of 1.968(1) Å for 3,3′-spirobis(3-selenanaphthalide) [44] and significantly longer than that observed in 2-carboxylphenylmethyl selenoxide [45], {Se–O (1.774 Å)}. The O1A–Se–O1A bond angle is 172.99° which is deviated from the linear arrangement of 180°. Interestingly, the O1A–Se–O1A bond angle is equal to the reported bond angle for the aliphatic dioxaselenanonane **4** (172.99°) [20], and in good agreement with the reported bond angle for aromatic analogue of 3,3′-spirobis(3-selenanaphthalide) **6** (172.4°) [44]. The dihedral angle between the planes defining the two chelate rings *i.e.*, O1A–Se–C1A–C6AC7A and Se–O1A–C7A–C6A–C1A is 75.58. The packing diagram of compound **20** shows weak intermolecular O···Se and O···H interactions with distances of 3.053(2) and 2.610(2) Å, respectively to form a two dimensional supramolecular network (Figure 6). The intermolecular O2A···H3 bond distance is 2.609(2) Å with bond angle 157.7(3)°.

#### 2.3.3. Molecular Structure of **21**

**Figure 7:**
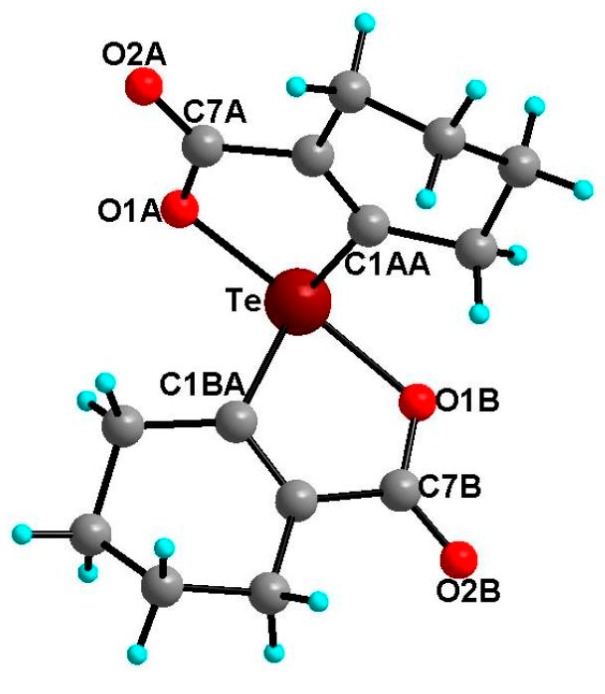
Molecular structure of compound **21**. Selected bond lengths (Å) and bond angles (°); Te–C1AA 2.055(1), Te–C1BA 2.138(12), Te–O1A 2.114(3), Te–O1B 2.082(3), O2A–C7A 1.214(5), O2B–C7B 1.205(5), O1A–Te–O1B 161.71(1), C1AA–Te–C1BA 98.6(5).

**Figure 8:**
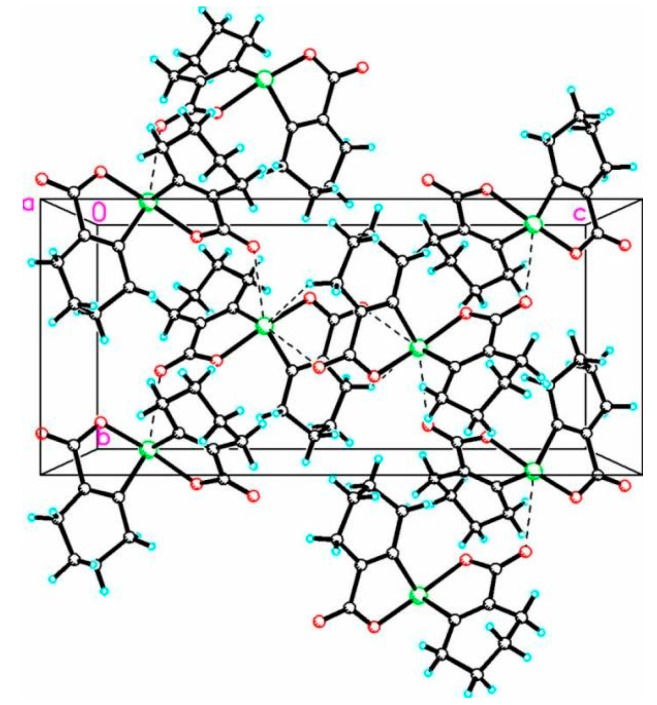
Two-dimensional packing diagram of compound **21**. Selected bond lengths (Å) and bond angles (°); Te···O2A 2.936(35) Å, Te···O2A–C7A 126.4(29)°.

Compound **21** (Figure 7) crystallizes in orthorhombic crystal system with *P*bca space group. The geometry around the tellurium atom is distorted trigonal bipyramidal and is very similar to the selenium analogue **20** in which both Te–1OA and Te–O1B occupy the axial positions, while Te–C1BA, Te–C1AA and the lone pair occupy the equatorial positions. The Te–O1A and Te–O1B bond lengths are 2.114(3) and 2.082(3) Å, respectively. These distances are close to the sum of covalent radii of tellurium and oxygen (2.04 Å) [38]. The O1A–Te–O1B bond angle in **21** is 161.7(1)° which is similar to that reported for aromatic spirotellurane (161.3°) [34]. The dihedral angles between two planes *i.e.*, Te–C1AA–C6AA–C7A–O1A and Te–O1B–C7B–C6BA–C1BA is 77.66(21). The crystal packing diagram shows intermolecular Te···O (carbonyl) interaction (Figure 8).

### 2.4. Glutathione Peroxidase-Like Activity

The glutathione peroxidase activity of the compounds was determined by the coupled reductase assay [46]. In this assay, the GPx-like activity was measured by a coupled enzyme containing nicotinamide adenine dinucleotide phosphate (NADPH) (0.01 M), glutathione (GSH) (0.05 M), catalysts (0.002 M), H_2_O_2_ (0.026 M), glutathione reductase (GR) (1.3 unit). The reduction of H_2_O_2_ by GSH was also recorded using ebselen for comparison. The decrease in NADPH concentration was monitored spectrophotometrically at 340 nm and results are summarized in Table 1. It was found that di-(*o*-formylcyclohex-1-ene)diselenide (**16**) is more efficient catalyst in comparison with ebselen, (2-phenyl-1,2-benzisoselenazol-3(2*H*)-one) [47], and bis(*o*-formylphenyl)diselenide [24]. The higher activity of **16** is probably due to the presence of stronger Se···O intramolecular interaction in comparison with bis(*o*-formylphenyl)diselenide. This stabilizes the selenenyl sulfide intermediate which in turn reacts with thiol to produce the disulfide. However, **17** and **19** do not show significant activity. The spirocyclic derivatives **20** and **22** where Se is in +4 oxidation state also did not show any significant activity. A similar result has been reported by Singh and co-workers [22] and Back and co-workers [23] in the case of aromatic spirocyclic derivatives. The reaction rates were similar to the control reaction rate. The GPx-like activity of compound **16** was calculated to be V_0_ = 49.8 ± 1.61 μM min^−1^ which is better than that of bis(*o*-formylphenyl)diselenide and standard ebselen (Table 1).

**Table 1:** Glutathione peroxidase-like activity of organoselenium compounds determined by coupled reductase assay.

Compound *	Average (V_0_) μM min^−1^
Control	10.5 ± 1.06
Ebselen [47]	27.8 ± 1.99
Bis(*o*-formylphenyl)diselenide [24]	34.3 ± 1.91
16	49.8 ± 1.61
17	9.4 ± 0.51
19	6.8 ± 0.77
20	16.4 ± 2.6
22	8.4 ± 0.24

*: The initial rates were calculated by following the depletion of NADPH by UV method at 25 °C. The reactions were carried out in 100 mM phosphate buffer, pH 7.48, with EDTA (1 mM), NADPH (0.01 M), GSH (0.05 M), catalysts (0.002 M), H_2_O_2_ (0.026 M), GR (1.3 unit).

#### 2.4.1. Mechanistic Studies on Di-(2-formylcyclohexenyl)diselenide **16**

To understand the mechanism and identify the intermediates involved in the catalytic reaction, ^77^Se-NMR spectroscopy has been used since its chemical shift is very sensitive to the environment. The three major intermediates involved in the peroxidase reduction, *i.e.*, RSeH, RSeOH and RSeSPh, are expected to show large differences in ^77^Se-NMR chemical shift values.

#### 2.4.2. Reaction of **16** with PhSH Followed by TBHP

The reaction of di-(2-formylcyclohexenyl)diselenide **16** with 3 equivalents of PhSH (thiophenol) does not show any new peak corresponding to the formation of the expected selenenyl sulfide **24** even after 30 min. It indicates that the reactivity of PhSH towards **16** is very slow. The addition of *tert*-butylhydroperoxide (TBHP) to the above mixture results in the instantaneous formation of the selenenyl sulfide **24** (indicated by ^77^Se-NMR signal at 705 ppm). The signal is shifted downfield in comparison to the reported value of diselenide **3** (545 ppm) [22]. This significant downfield shift is due to the existence of strong Se···O intramolecular interaction in selenenyl sulfide **24** which suggests that **24** is very stable in solution and is not disproportionated to the corresponding **16**.

#### 2.4.3. Reaction of Di-(2-formylcyclohexenyl)diselenide **16** with TBHP Followed by PhSH

In the ^77^Se-NMR spectrum, four signals at 488, 1393, 1379, and 1338 ppm were observed for the reaction of **16** with two equivalents of TBHP. These correspond to diselenide **16**, selenenic acid (RSeOH) **25**, seleninic acid (RSeO_2_H) **26** and selenonic acid (RSeO_3_H) **27,** respectively. Similar intermediates have been observed for the reaction of bis[2-(4,4-dimethyl-2-oxazolinyl)phenyl]diselenide [17] with H_2_O_2_. The ^77^Se-NMR chemical shifts are significantly downfield shifted compared with that of selenenic acid (1206 ppm), seleninic acid (1149 ppm) and selenonic (1138 ppm) formed during the reaction of (bis[2-(4,4-dimethyl-2-oxazolinyl phenyl]diselenide with an excess of H_2_O_2_ and also downfield shifted in comparison to the selenenic acid (1200 ppm) of **3** [22].


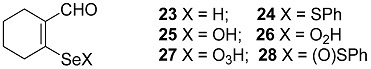


**Scheme 3:**
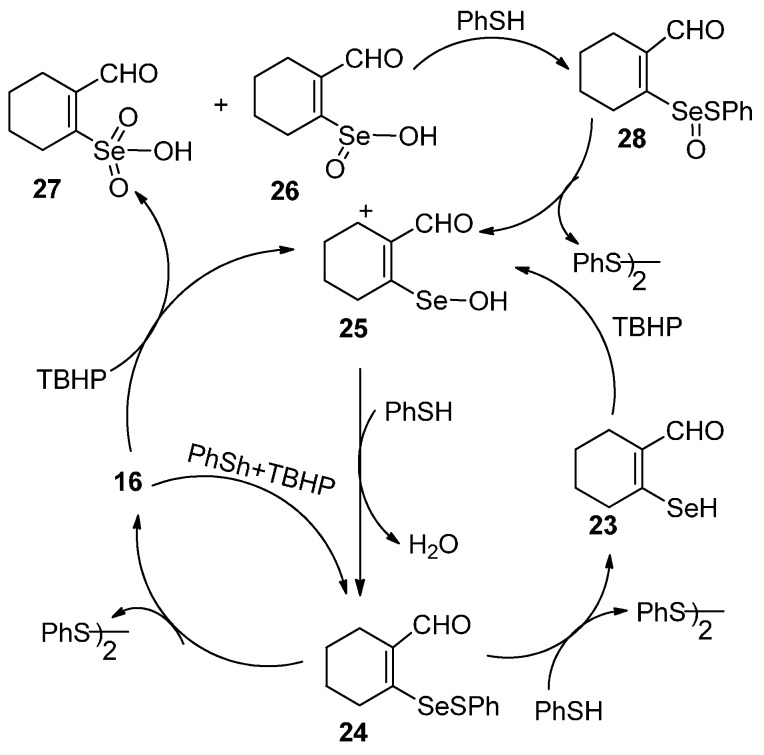
GPx activity of di-(2-formylcyclohexenal)diselenide (**16**).

When the reaction mixture containing TBHP was treated with seven equivalents of PhSH, peaks corresponding to the intermediates selenenic acid **25**, seleninic acid **26** and selenonic acid **27** disappeared. Two new peaks at 705 and 866 ppm along with peak for **16** were observed. The peaks at 705 and 866 ppm probably correspond to the selenenyl sulfide **24** and selenoxysulfide **28**, respectively. Selenoxysulfide **28** is formed by the reaction of selenenic acid **25** with PhSH. The reaction of selenenic acid with PhSH is analogous to the observation by Mugesh *et al.* [17] where bis[2-(4,4-dimethyl-2-oxazolinyl)phenyl]diselenide reacts rapidly with thiol to give corresponding selenoxysulfide intermediates. After the addition of 14 equivalents of PhSH, peaks corresponding to the **16** and other intermediates had disappeared except for the peak of selenenyl sulfide **24**. Based on these observations and mechanisms reported for related diselenides, the following mechanism for the catalytic action of **16** (Scheme 3) is proposed.

## 3. Experimental Section

### 3.1. General Information

All the organochalcogen derivatives were synthesized under nitrogen or argon atmosphere using standard Schlenk line techniques. Solvent were purified and dried by standard procedures and were freshly distilled prior to use [48]. All the chemicals used were reagent grade and were used as received. Melting points were recorded in capillary tubes. The NMR spectra were recorded in CDCl_3_ solvent. The ^1^H (400 MHz), ^13^C (100 MHz), ^77^Se (57.26/76.4 MHz) and ^125^Te (126.3 MHz) spectra were recorded on a Varian Mercury plus or Bruker 400 MHz spectrometer. Chemical shifts cited were referenced with respect to TMS for (^1^H and ^13^C) as internal standard and Me_2_Se (for ^77^Se), Me_2_Te (for ^125^Te) as external standards. Elemental analysis was performed on Carlo-Erba model 1106 and Eager 300 EA112 elemental analyzers. The IR spectra were recorded in the range 400–4000 cm^−1^ by using KBr pellets for solid samples on a Thermo Nicolet Avatar 320 FT-IR spectrometer. Mass spectral (MS) studies were completed by using a QTOF Micro mass spectrometer with electrospray ionization mode analysis. In the case of isotopic patterns, the value is given for the most intense peak. The UV–VIS spectra in solution for GPx activity were recorded with a JASCO, V-570 spectrometer.

The single crystal X-ray diffraction measurements for compounds were performed on Oxford Diffraction Gemini diffraction measurement device with graphite monochromated Mo Kα radiation (λ = 0.7107 Å). The structures were determined by routine heavy-atom method using SHELXS 97 [49] and refined by full-matrix least-squares with the non-hydrogen atom anisotropic and hydrogen atoms with fixed isotropic thermal parameters of 0.07 Å by means of SHELXS 97 program [50]. The structure refinement parameters for compounds **19**–**21** are given in Table 2. CCDC-1041184 (**19**), CCDC-1041181 (**20**), CCDC-1041182 (**21**), contain the supplementary crystallographic data for this paper. These data can be obtained free of charge from The Cambridge Crystallographic Data Centre via www.ccdc.cam.ac.uk/data_request/cif.

**Table 2:** Crystal and structure refinement data for **19**–**21**.

Compound	19	20	21
Empirical formula	C_14_H_22_O_2_Se	C_56_H_64_O_16_Se_4_	C_14_H_16_O_4_Te
Formula weight	301.28	1308.91	375.87
Crystal system	Monoclinic	Monoclinic	Orthorhombic
Space group	*P*2_1_/c	*C*2/c	*P*bca
*a* (Å)	8.7358(12)	15.0340(13)	15.7046(2)
*b* (Å)	9.1761(12)	8.5925(7)	8.83038(16)
*c* (Å)	19.689(2)	10.4102(8)	19.1603(3)
α (°)	90	90	90
β (°)	116.096(8)	112.358(4))	90
γ (°)	90	90	90
*V* (Å^3^)	1417.4(3)	1242.69(18)	2657.11(8)
*Z*	4	1	8
D (calcd) (Mg/m^3^)	1.412	1.748	1.879
Absorption coefficient (mm^−1^)	2.639	2.027	17.759
Reflections collected	7290	11304	5111
Final *R*(F) [*I* > 2σ(*I*)] ^a^	0.0567	0.0237	0.0515
*wR*(F2) indices [*I* > 2σ(*I*)]	0.1414	0.0597	0.1361
Data/restraints/parameters	2522/17/158	1963/0/87	2513/72/210
Goodness-of-fit on F^2^	1.024	1.099	1.032

^a^: *R*(*F*o) = ∑||*F*o| − |*F*c||/∑|*F*o| and *wR*(*F*o^2^) = {∑[*w*(*F*o^2^ − *F*c^2^)^2^]/∑[*w*(*F*c^2^)^2^}^1/2^.

### 3.2. Synthesis

*Di(2-hydroxymethylcyclohex-1-ene)selenide* **19**. To a stirred solution of di-(2-formylcyclohexenyl)selenide **17** (0.106 g, 0.34 mmol) in dry methanol (5 mL) was added an excess of NaBH_4_ (0.051 g, 1.36 mmol) at 0 °C. The reaction mixture was stirred for 6 h. Solvent was evaporated and the residue poured into crushed ice. The product was extracted with chloroform and the combined organic layers were washed with a saturated brine solution. It was dried over sodium sulfate and concentrated to give **19** as white solid.


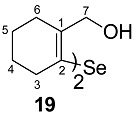


Yield: 0.060 g (25%), mp 126–128 °C; ^1^H-NMR (400 MHz, CDCl_3_): δ 4.30 (s, 4H), 2.29–2.27 (dd, 8H), 1.65–1.64 (dd, 8H); ^13^C-NMR (CDCl_3_): δ 141.2 (C1), 126.7 (C2), 66.4 (C7), 34.4 (C3), 29.4 (C6), 24.4 (C4), 22.7 (C5); ^77^Se-NMR (CDCl_3_): δ 325. ES-HRMS: *m*/*z* calcd. for C_14_H_22_SeO_2_: 302.0785 [M]^+^; found: 325.0688 [M + Na]^+^. FT-IR υ 1019, 1429, 1443, 2875, 2855, 2926, 3298 cm^−1^. Anal. Calcd. for C_14_H_22_SeO_2_: C, 55.81; H, 7.36; found C, 56.18; H, 6.97.

*Spirodioxyselenurane* **20**. To a stirred solution of di-(2-formylcyclohexenyl)selenide **17** (0.112 g, 0.34 mmol) in dry CH_2_Cl_2_ (10 mL) was added 30% of H_2_O_2_ (120 μL, 1.02 mmol) at 0 °C. The reaction mixture was stirred for 24 h at room temperature to give compound **20** as a white solid.


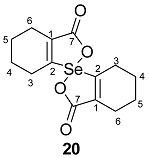


Yield: 0.05 g (48%). mp 87–89 °C. ^1^H-NMR (400 MHz, CDCl_3_): δ (ppm) 2.57–2.50 (m, 4H), 2.39–2.38 (m, 8H), 1.86–1.67 (m, 8H); ^13^C-NMR (CDCl_3_): δ 190.3 (C7), 150.1 (C2), 140.1 (C1), 30.5 (C6), 20.5 (C4), 20.4 (C3), 20.2 (C5); ^77^Se-NMR (CDCl_3_): δ 801; ES-HRMS: *m*/*z* calcd. for C_14_H_16_SeO_4_: 328.0214 [M]^+^; found: 329.0294 [M + H]^+^. FT-IR (KBr) υ 740, 759, 1138, 1238, 1683, 2941cm^−1^. Anal. Calcd. for C_14_H_16_SeO_4_: C, 51.39; H, 4.93; found C, 50.84; H, 4.81.

*Spirodioxytellurane* **21**. A solution of compound **18** (0.1 g, 0.28 mmol) in dry CH_2_Cl_2_ (5 mL) was treated with an aqueous 30% of H_2_O_2_ (99 μL, 0.86 mmol) at 0 °C. The mixture was stirred for 24 h at room temperature; the solvent was evaporated to give compound **21** as a white solid.


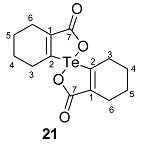


Yield: 0.09 g (25%), mp 95–97 °C; ^1^H-NMR (400 MHz, CDCl_3_): δ (ppm) 1.84–1.74 (m, 8H), 2.76–2.23 (m, 8H); ^13^C-NMR (CDCl_3_): δ 190.3 (C7), 150.2 (C2), 140.2 (C1), 30.6 (C6), 20.5 (C3), 20.40 (C4), 20.2 (C5); ^125^Te-NMR (CDCl_3_): δ 1088.7. ES-HRMS: *m*/*z* calcd. for C_14_H_16_TeO_4_ (378.0111) [M]^+^; found 379.0174 [M + H]^+^. FT-IR (KBr) υ 702, 975, 1203, 1584, 1663, 2931cm^−1^. Anal. Calcd. for C_14_H_16_TeO_4_: C, 44.74; H, 4.29; found C, 45.02; H, 5.38.

*Spirodioxyselenurane* **22**. To a solution of selenide **19** (0.1 g, 0.28 mmol) in dry CH_2_Cl_2_ (5 mL) was added an aqueous 30% of H_2_O_2_ (99 μL, 0.86 mmol) at 0 °C. The resultant mixture was stirred for 24 h at room temperature, the solvent was evaporated to afford **22** as a white solid.


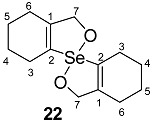


Yield: 0.07 g (49%). mp 82–84 °C. ^1^H-NMR (400 MHZ, CDCl_3_): δ (ppm) 4.66–4.55 (d, 4H, ^2^*J* = 12 Hz), 2.52–2.06 (m, 8H), 1.77–1.67 (m, 8H); ^13^C-NMR (CDCl_3_): δ; 144.3 (C2), 131.9 (C1), 76.1 (C7), 25.5 (C6), 25.3 (C4), 24.3 (C5), 21.5 (C3); ^77^Se-NMR (CDCl_3_): δ 908; ES-HRMS: *m*/*z* calcd. for C_14_H_20_SeO_2_: 300.0629, [M]^+^; found: 301.0705 [M + H]^+^. Anal. Calcd. for C_14_H_20_SeO_2_: C, 56.19; H, 5.74; found C, 50.60; H, 5.99.

### 3.3. Coupled Reductase Assay

The GPx-like activities of **16**–**20** were measured by JASCO spectrophotometer according to the literature method using ebselen as the standard. The catalytic reaction was carried out at room temperature (25 °C) in 1 mL of the solution containing 100 mM potassium phosphate buffer, pH 7.5, 1 mM EDTA, 0.1 mM GSH, 0.25 mM of NADPH, 0.020 mM of catalyst and 0.26 mM of H_2_O_2_. The activity was followed by the decrease of NADPH on addition of H_2_O_2_ and absorption was measured at 340 nm (ε_max_ = 6.22 × 10^3^ M^−1^ cm^−1^) (chemical reactions (1)–(3)). Each of the experiments was carried out in triplicate.


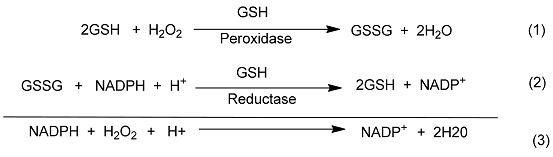


## 4. Conclusions

Di-(2-formylcyclohexenyl)diselenide **16** showed better GPx like activity than the aromatic analogue bis(*o*-formylphenyl)diselenide whereas di(2-hydroxymethylcyclohex-1-ene)selenide **19** and spirodioxyselenurane **20** and **22** did not show any significant activity.

## Supplementary Materials

Supplementary materials can be accessed at: http://www.mdpi.com/1420-3049/20/07/12670/s1.
