# Supplementary material for: Synthesis, Structure and Antioxidant Activity of Cyclohexene-Fused Selenuranes and Related Derivatives
Source: Molecules. 2015 Jul 13;20(7):12670–85. doi: 10.3390/molecules200712670 (PMC6332442; doi:10.3390/molecules200712670)
Supplement: Supplementary file 1 [file molecules-20-12670-s001.zip › molecules-85709-supplementary-final-done.pdf]

Supporting Information

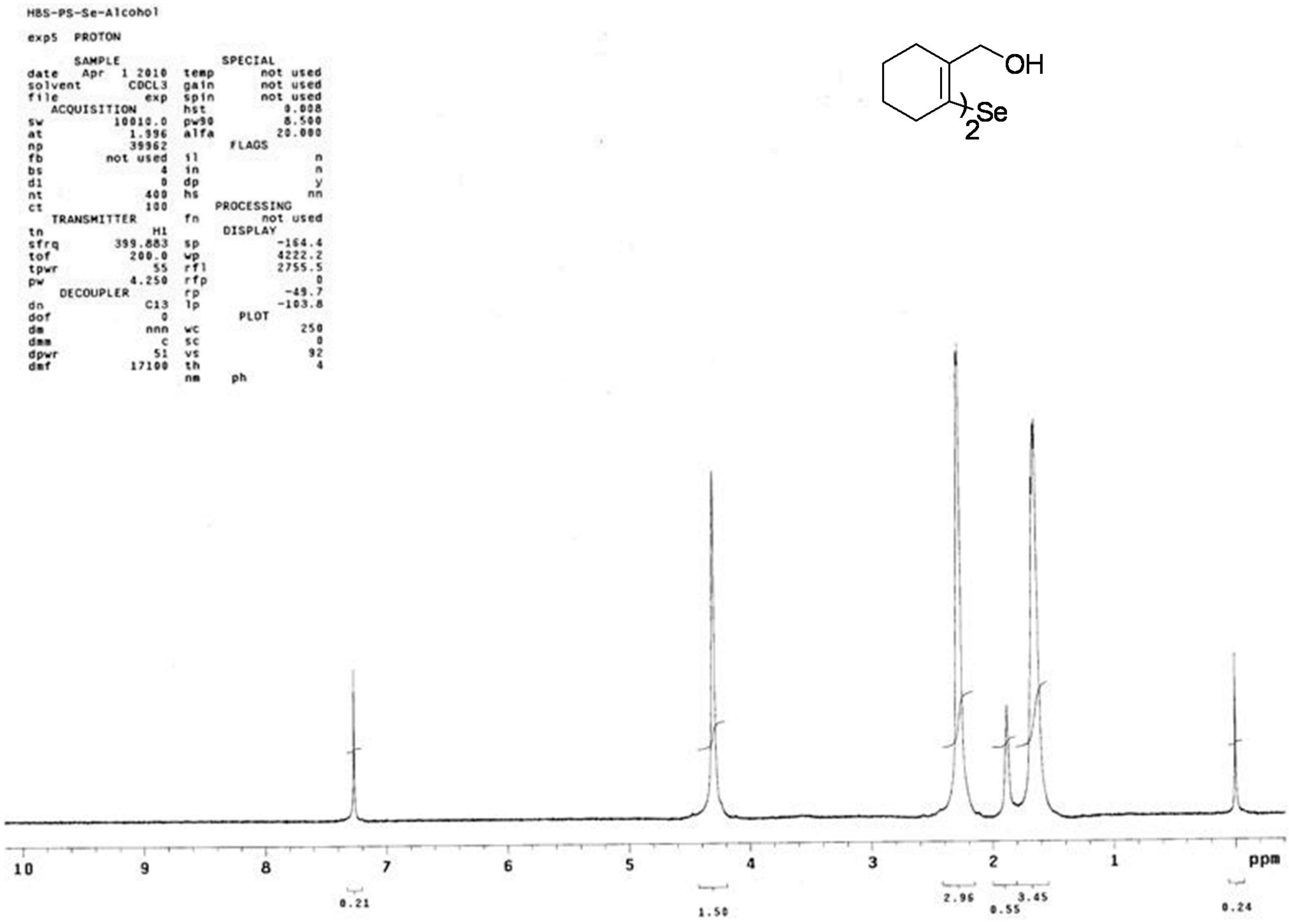

Figure S1. ^{1}H-NMR spectrum of **19** ($CDCl_3$).

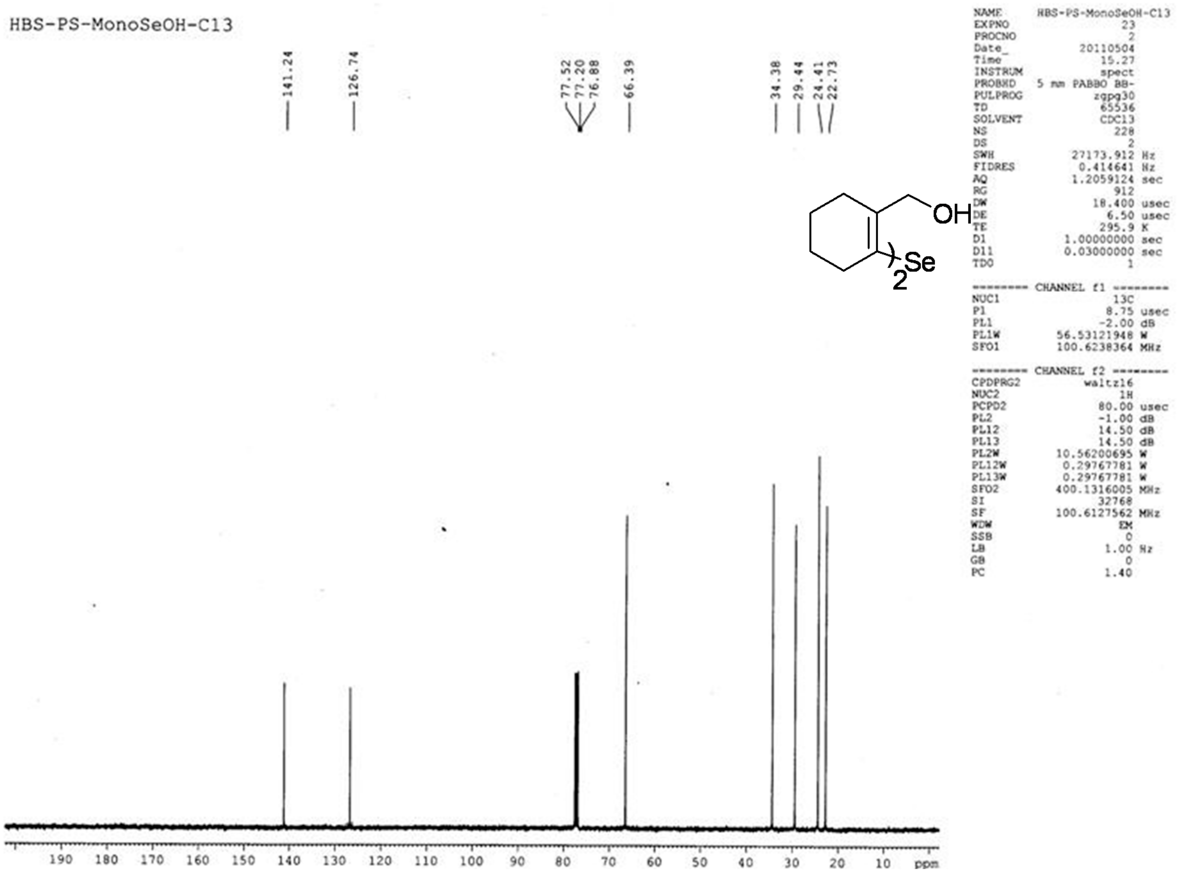

Figure S2. ^{13}C-NMR spectrum of **19** ($CDCl_3$).

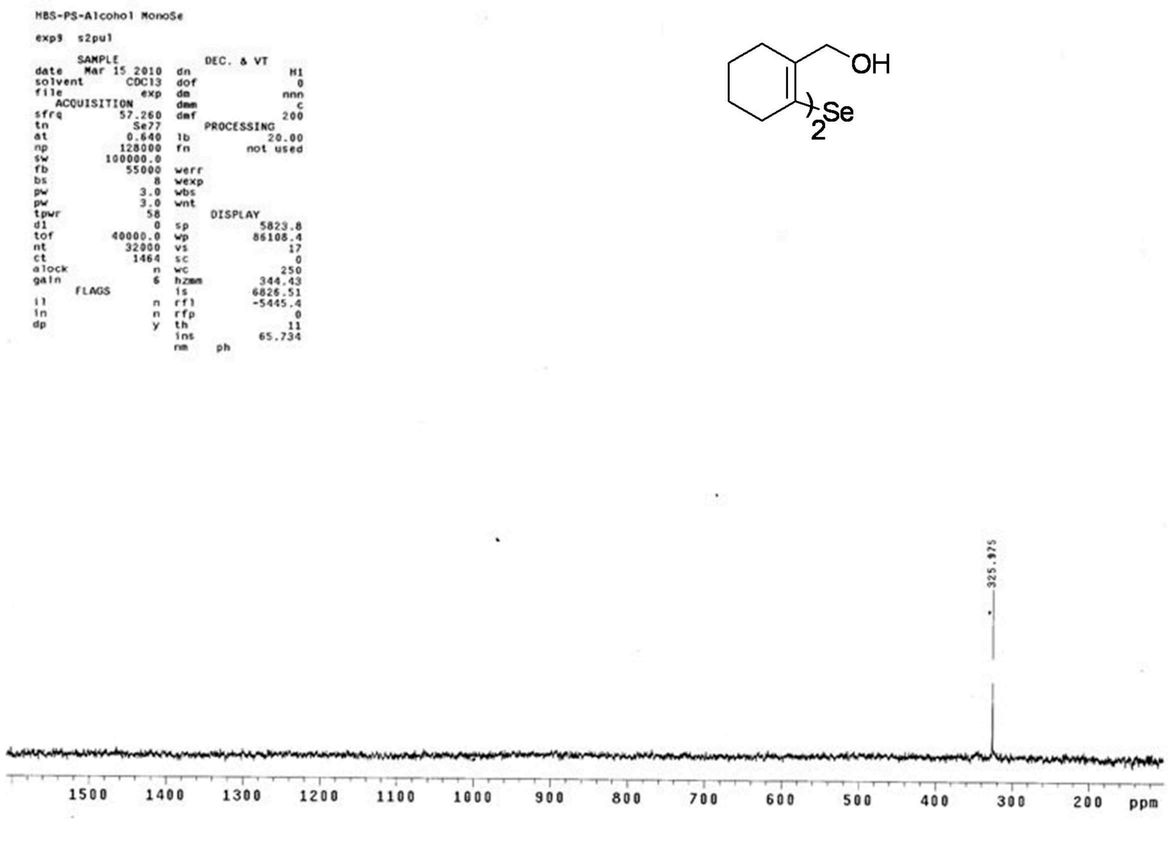

Figure S3. ^{77}Se-NMR spectrum of **19** ($CDCl_3$).

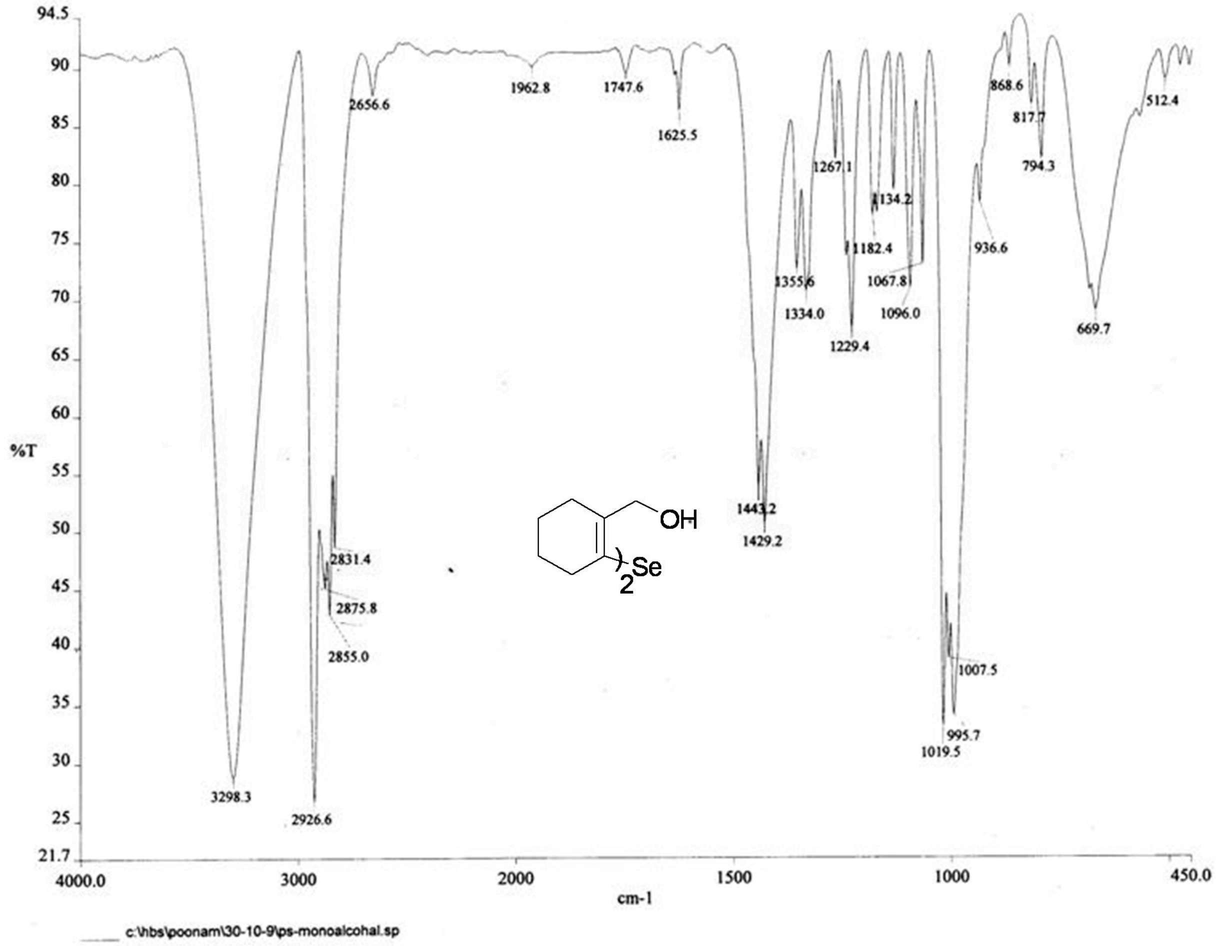

Figure S4. FT-IR spectrum of **19**.

Elemental Composition Report

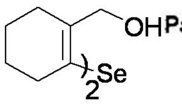

Page 1

Single Mass Analysis (displaying only valid results)
Tolerance = 10.0 PPM / DBE: min = -1.5, max = 50.0
Isotope cluster parameters: Separation = 1.0 Abundance = 1.0%

Monoisotopic Mass, Odd and Even Electron Ions
22 formula(e) evaluated with 1 results within limits (up to 50 closest results for each mass)

Micromass : Q-Tof micro (YA-105) Dept. Of Chemistry I.I.T.(B) 27-May-201112:12:03

C14H22O2Se
HBS-PS-ALCOHOL SE 83 (0.822) AM (Cen,5, 80.00, Ht,5000.0,556.28,1.00); Sm (Md, 6.00); Sb (5,40.00); Cm (58:83) TOF MS ES+ 2.75e3

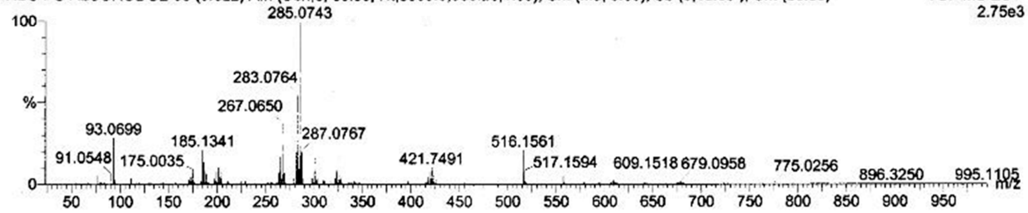

Minimum:				-1.5		
Maximum:		200.0	10.0	50.0		
Mass	Calc. Mass	mDa	PPM	DBE	Score	Formula
325.0688	325.0683	0.5	1.7	4.5	1	C14 H22 O2 Se Na

Figure S5. HRMS spectrum of **19**.

Eager 300 Report

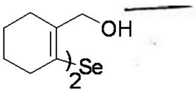

Page: 1 Sample: PS1 (PS1)

Method Name : SP061109
Method File : D:\CHNS2008\SP061109.mth
Chromatogram : PS1
Operator ID : SP
Analysed : 11/06/2009 09:03
Sample ID : PS1 (# 6)
Analysis Type : UnkNown (Area)

Company Name : C.E. Instruments
Printed : 11/6/2009 13:10
Instrument N. : Instrument #1
Sample weight : .654

Calib. method : using 'K Factors'

!!! Warning missing one or more peaks.

Element Name		%	Ret.Time	Area	BC	Area ratio	K factor
Carbon	55.81	56.1831	67	909526	RS	1.000000	.246719E+07
Hydrogen	7.36	6.9764	175	247576	RS	3.673724	.525645E+07
Totals		63.1596		1157102			

Figure S6. Elemental analysis (CHN) of **19**.

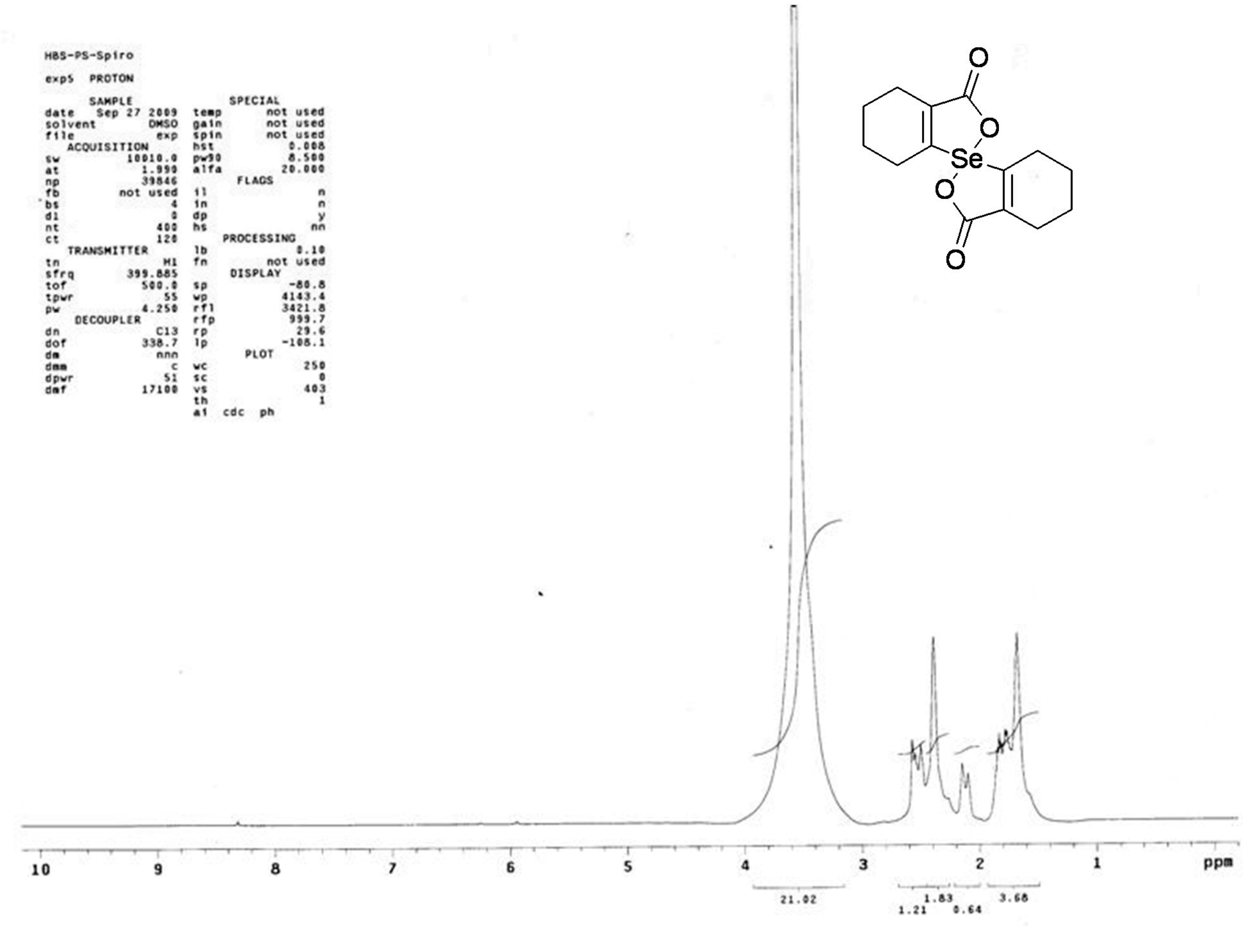

Figure S7. ^{1}H-NMR spectrum of **20** ($CDCl_3$).

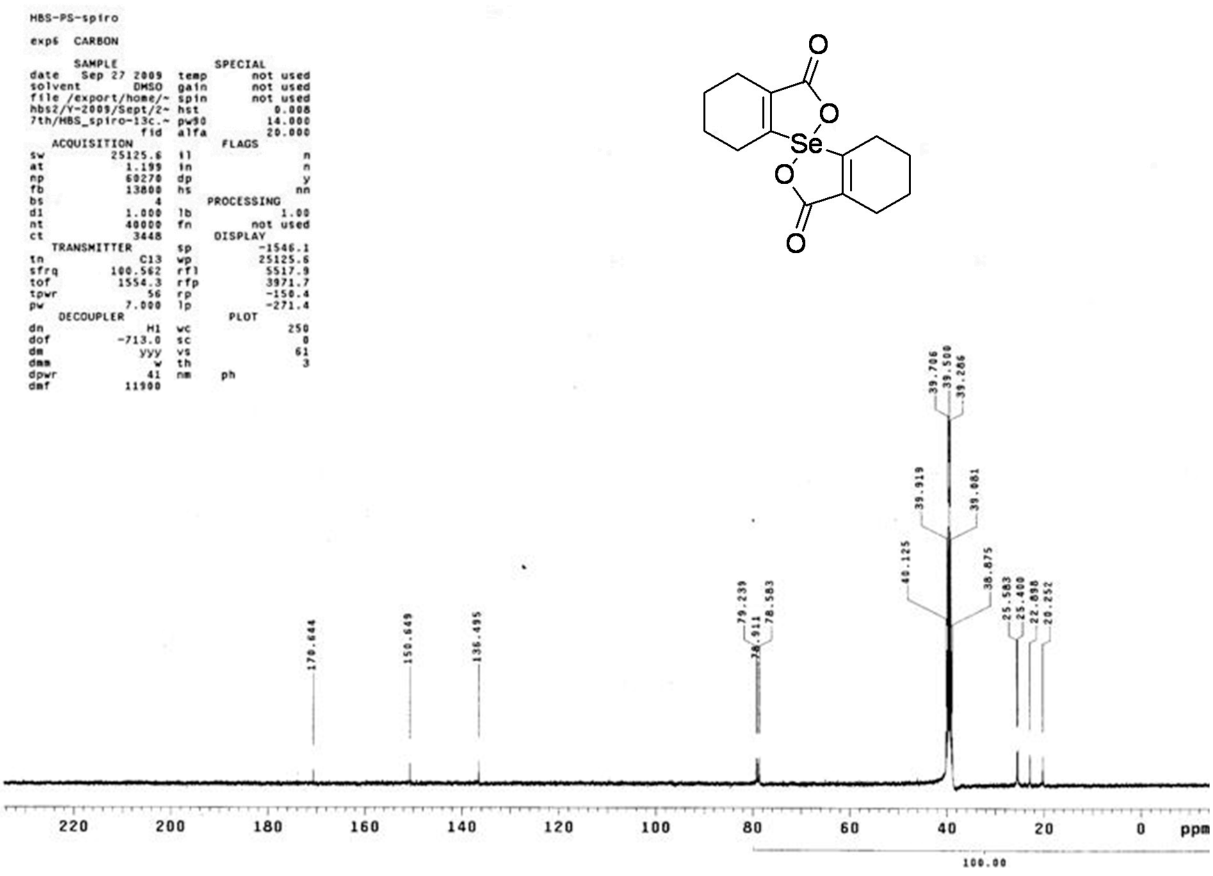

Figure S8. ^{13}C-NMR spectrum of **20** ($CDCl_3$).

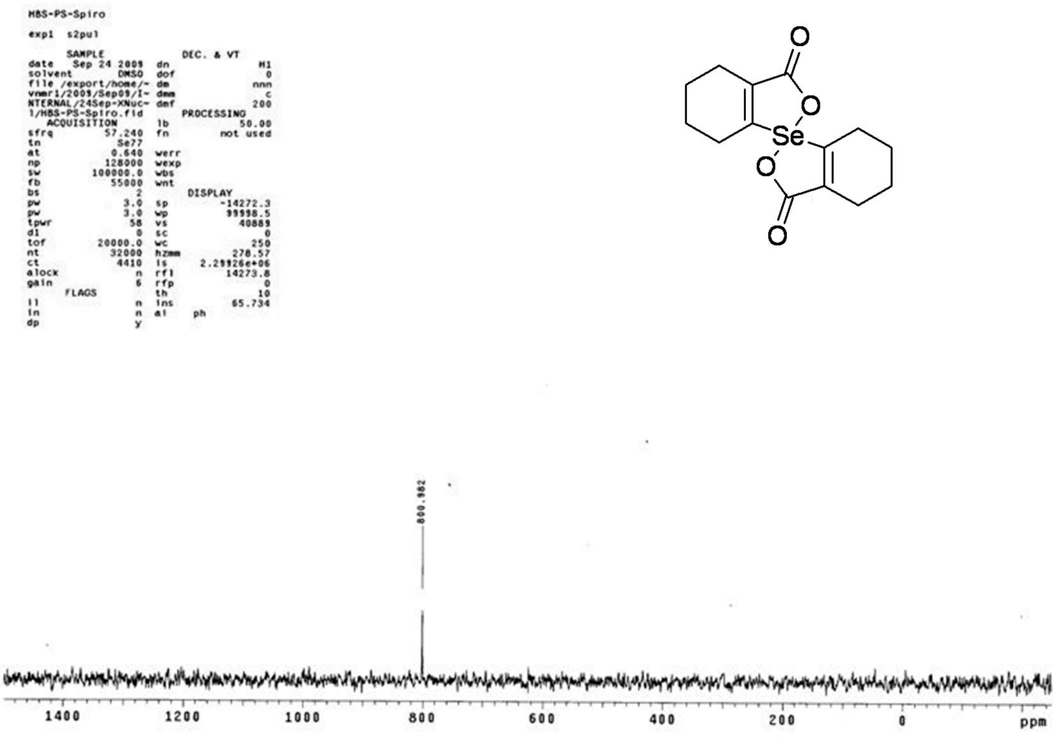

Figure S9. ^{77}Se-NMR spectrum of **20** ($CDCl_3$).

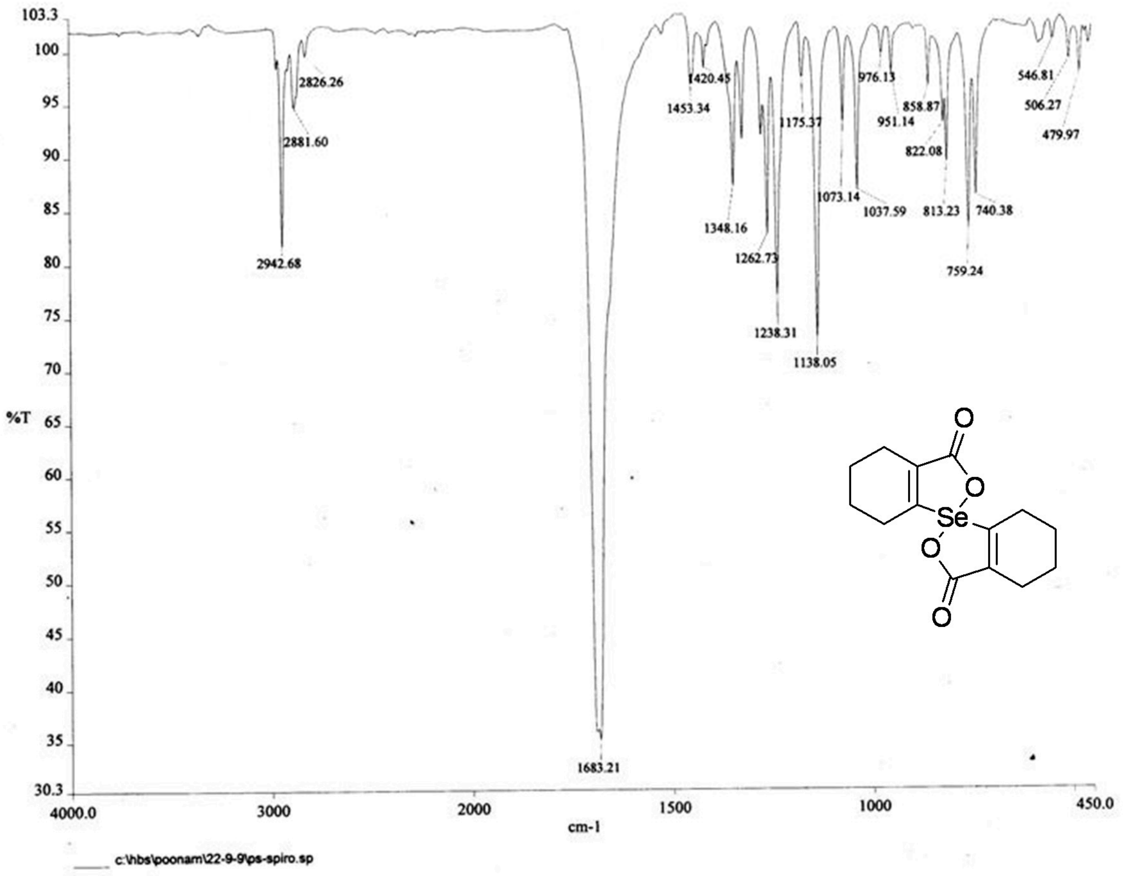

Figure S10. FT-IR spectrum of **20**.

Elemental Composition Report **Page 1**

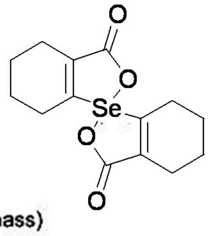

Single Mass Analysis (displaying only valid results)
Tolerance = 10.0 PPM / DBE: min = -1.5, max = 50.0
Isotope cluster parameters: Separation = 1.0 Abundance = 1.0%

Monoisotopic Mass, Odd and Even Electron Ions
9 formula(e) evaluated with 1 results within limits (all results (up to 1000) for each mass)

Micromass : Q-Tof micro (YA-105) Department Of Chemistry I.I.T.(B) 05-Aug-201015:19:45

C14H16O4Se
HBS-PS-SESPIRO 10 (0.186) AM (Top,4, Ht,5700.0,556.28,0.76); Sb (5,40.00); Cm (1:33) Ionisation Mode TOF MS ES+ 6.64e3

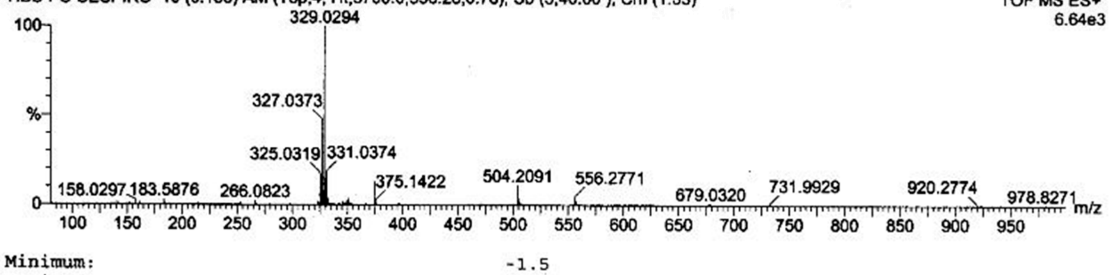

Minimum:				-1.5		
Maximum:		200.0	10.0	50.0		
Mass	Calc. Mass	mDa	PPM	DBE	Score	Formula
329.0294	329.0292	0.2	0.7	7.5	1	C14 H17 O4 Se

Figure S11. HRMS spectrum of **20**.

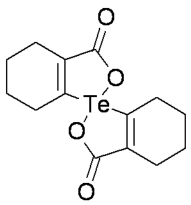

Eager 300 Report

Page: 1 Sample: TE-SPIRO (TE-SPIRO)

Method Name : SP280110
Method File : D:\CHNS2008\SP280110.mth
Chromatogram : TE-SPIRO
Operator ID : MP
Analysed : 01/28/2010 12:32
Sample ID : TE-SPIRO (# 12)
Analysis Type : UnkNown (Area)

Company Name : C.E. Instruments
Printed : 1/28/2010 14:59
Instrument N. : Instrument #1
Sample weight : 1.078

Calib. method : using 'K Factors'

!!! Warning missing one or more peaks.

Element Name		%	Ret.Time	Area	BC	Area ratio	K factor
Carbon	44.74	45.0227	67	1198946	RS	1.000000	.246135E+07
Hydrogen	4.29	5.3807	172	325334	RS	3.685276	.540442E+07
Totals		50.4034		1524280			

Figure S12. Elemental analysis (CHN) of **20**.

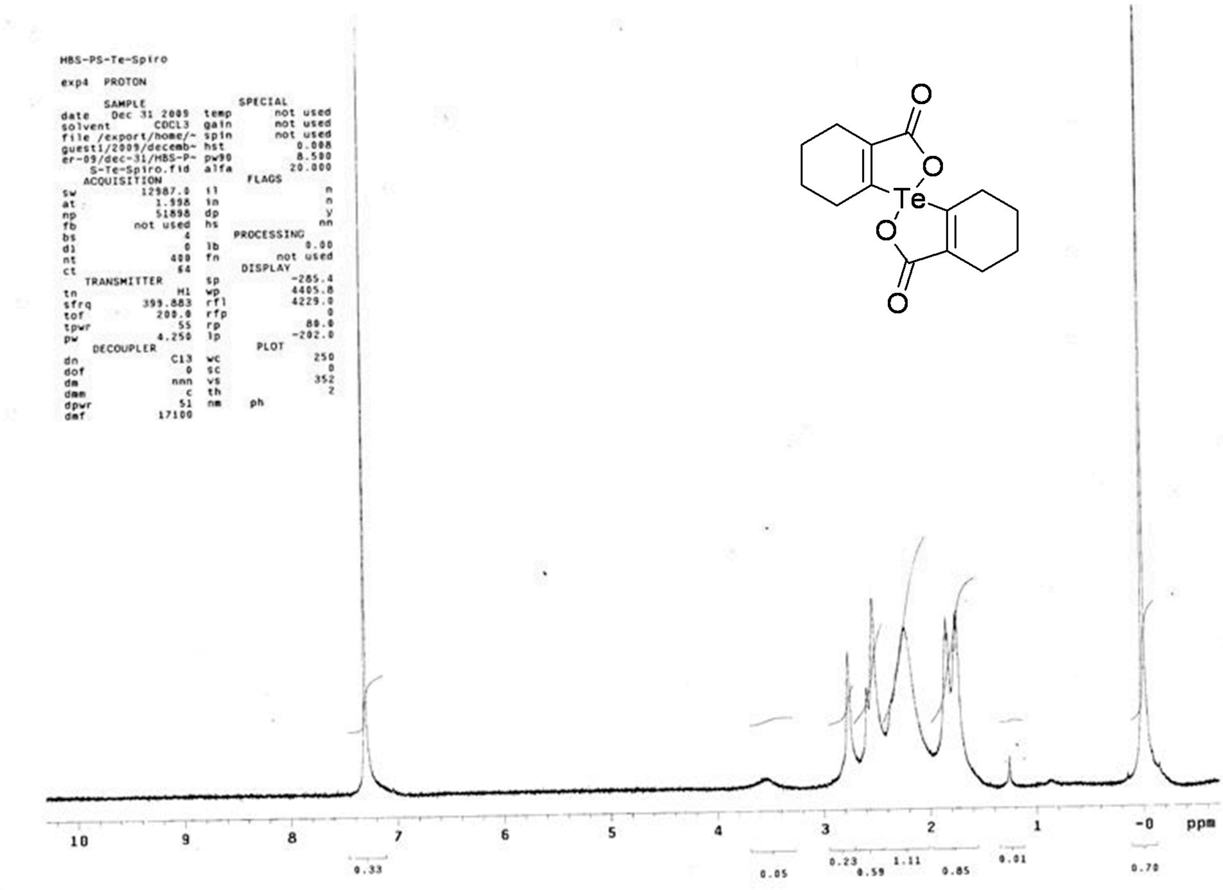

Figure S13. ^{1}H-NMR spectrum of **21** ($CDCl_3$).

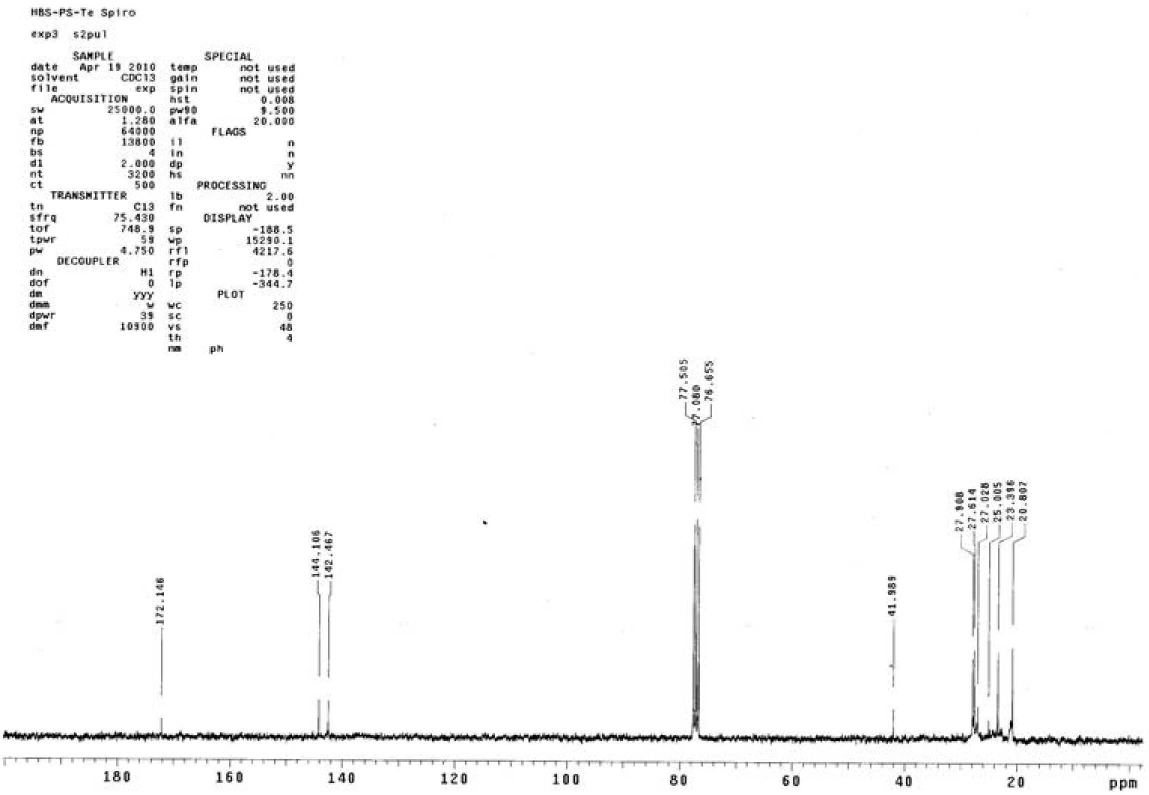

Figure S14. ^{13}C-NMR spectrum of **21** ($CDCl_3$).

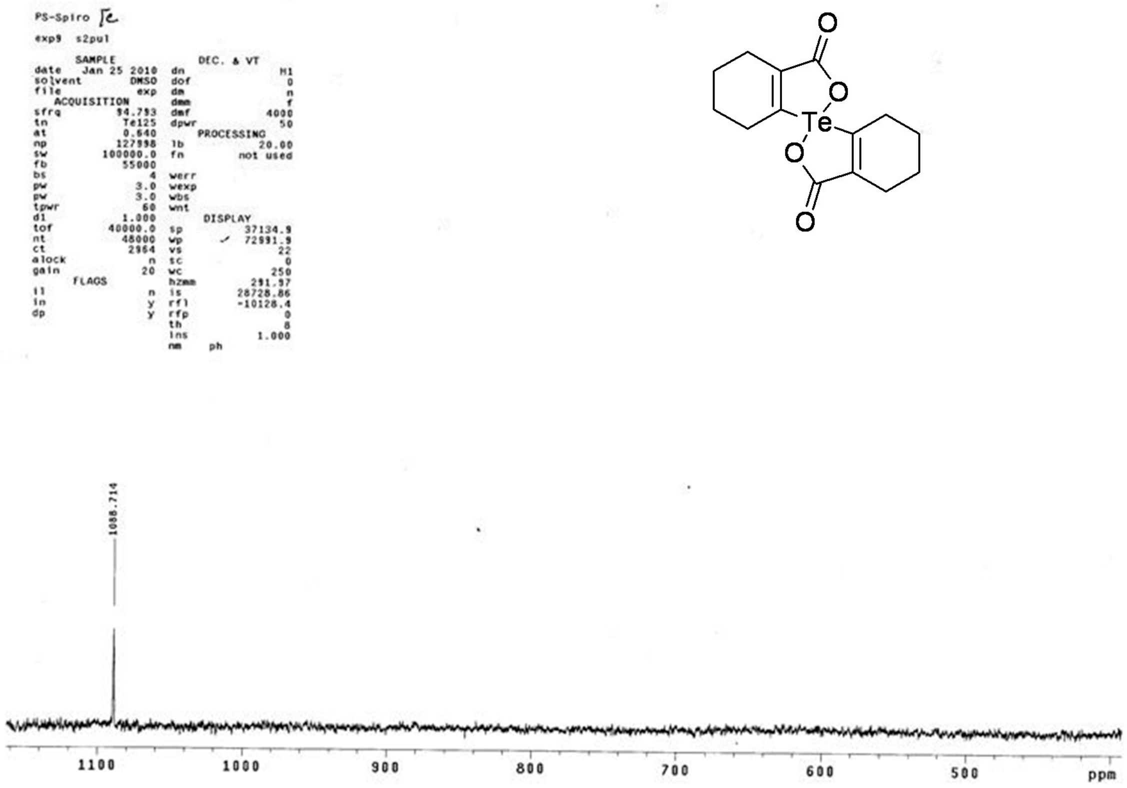

Figure S15. ^{125}Te-NMR spectrum of **21** ($CDCl_3$).

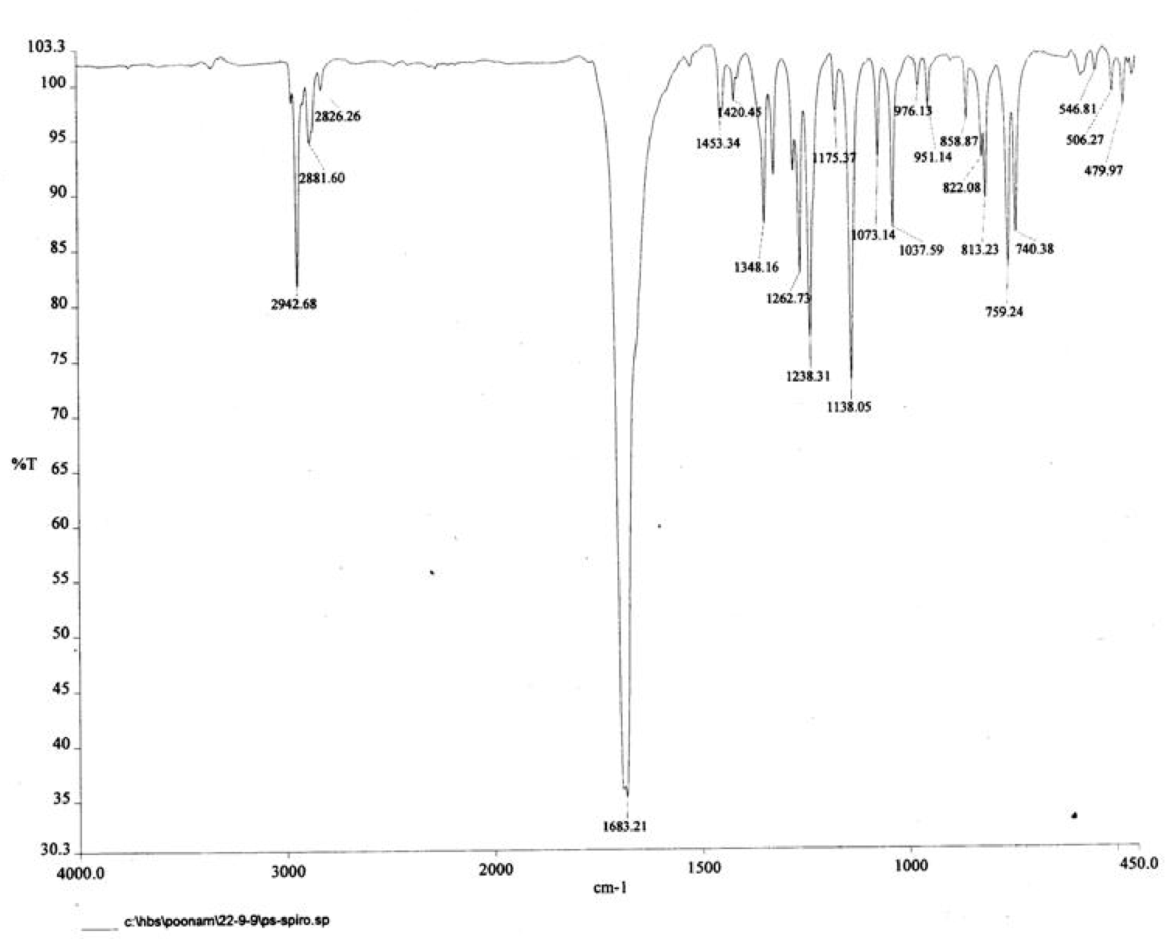

Figure S16. FT-IR spectrum of **21**.

Elemental Composition Report

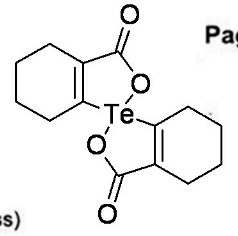

Page 1

Single Mass Analysis (displaying only valid results)

Tolerance = 10.0 PPM / DBE: min = -1.5, max = 50.0

Isotope cluster parameters: Separation = 1.0 Abundance = 1.0%

Monoisotopic Mass, Odd and Even Electron Ions

19 formula(e) evaluated with 1 results within limits (all results (up to 1000) for each mass)

Micromass : Q-Tof micro (YA-105) Department Of Chemistry I.I.T.(B) 05-Aug-201015:39:39

C14H16O4Te

HBS-PS-TESPIRO 29 (0.537) AM (Cen,4, 80.00, Ht,5700.0,556.28,0.76); Sm (SG, 2x4.00); Sb (5,40.00); Cm (1:56)

Ionisation Mode TOF MS ES+ 4.20e3

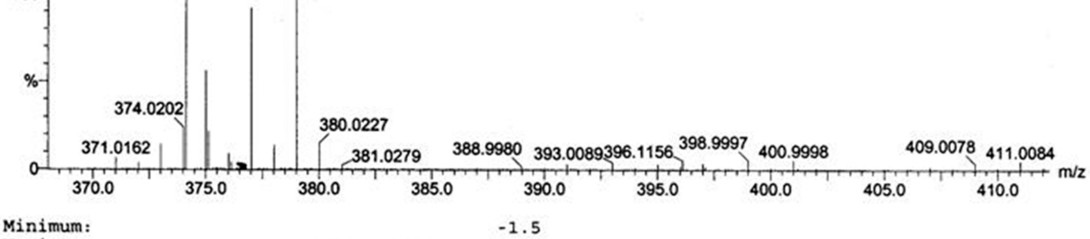

Minimum:				-1.5		
Maximum:		200.0	10.0	50.0		
Mass	Calc. Mass	mDa	PPM	DBE	Score	Formula
379.0174	379.0189	-1.5	-3.9	6.5	1	C14 H17 O4 Te

Figure S17. HRMS spectrum of **21**.

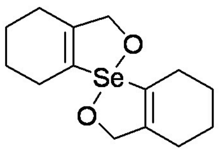

Eager 300 Report

Page: 1 Sample: HBSPSCHALC (HBSPSCHALC) H_2O_2

Method Name : SP040610
Method File : D:\CHNS2008\SP040610.mth
Chromatogram : HBSPSCHALC
Operator ID : SP
Analysed : 06/04/2010 13:49
Sample ID : HBSPSCHALC (# 16)
Analysis Type : UnkNown (Area)

Company Name : C.E. Instruments
Printed : 6/4/2010 15:45
Instrument N. : Instrument #1
Sample weight : .682

Calib. method : using 'K Factors'

!!! Warning missing one or more peaks.

Element Name	%	Ret.Time	Area	BC	Area ratio	K factor
Carbon	54.4053	67	1003864	RS	1.000000	.268468E+07
Hydrogen	7.1917	169	445628	RS	2.252695	.687010E+07
Totals	61.5970		1449492			

Figure S18. Elemental analysis (CHN) of **21**.

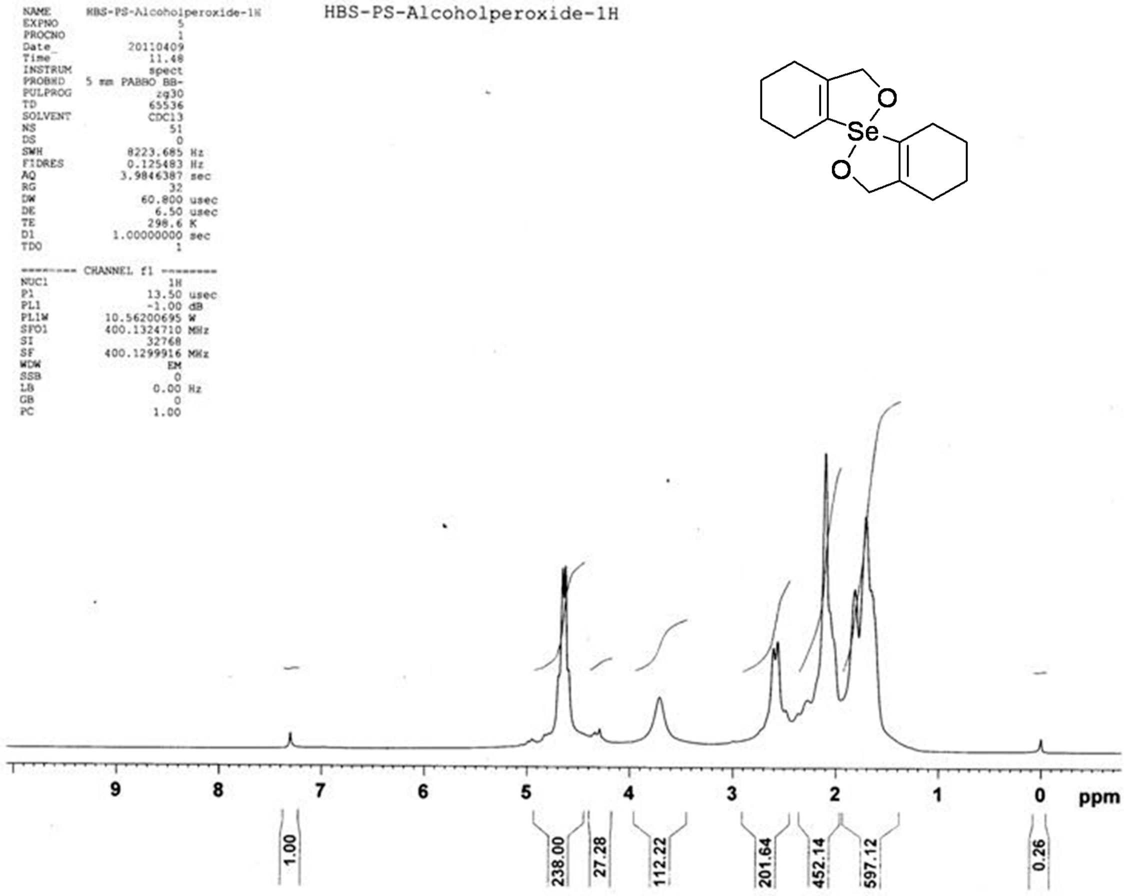

Figure S19. *Cont.*

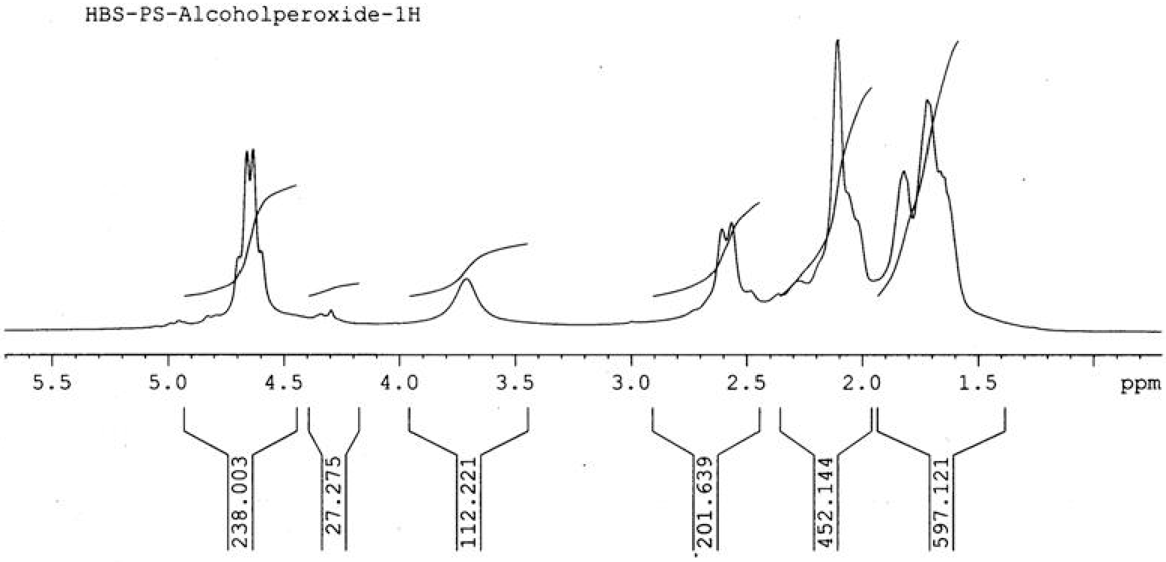

Figure S19. ^{1}H-NMR spectrum of **22** ($CDCl_3$).

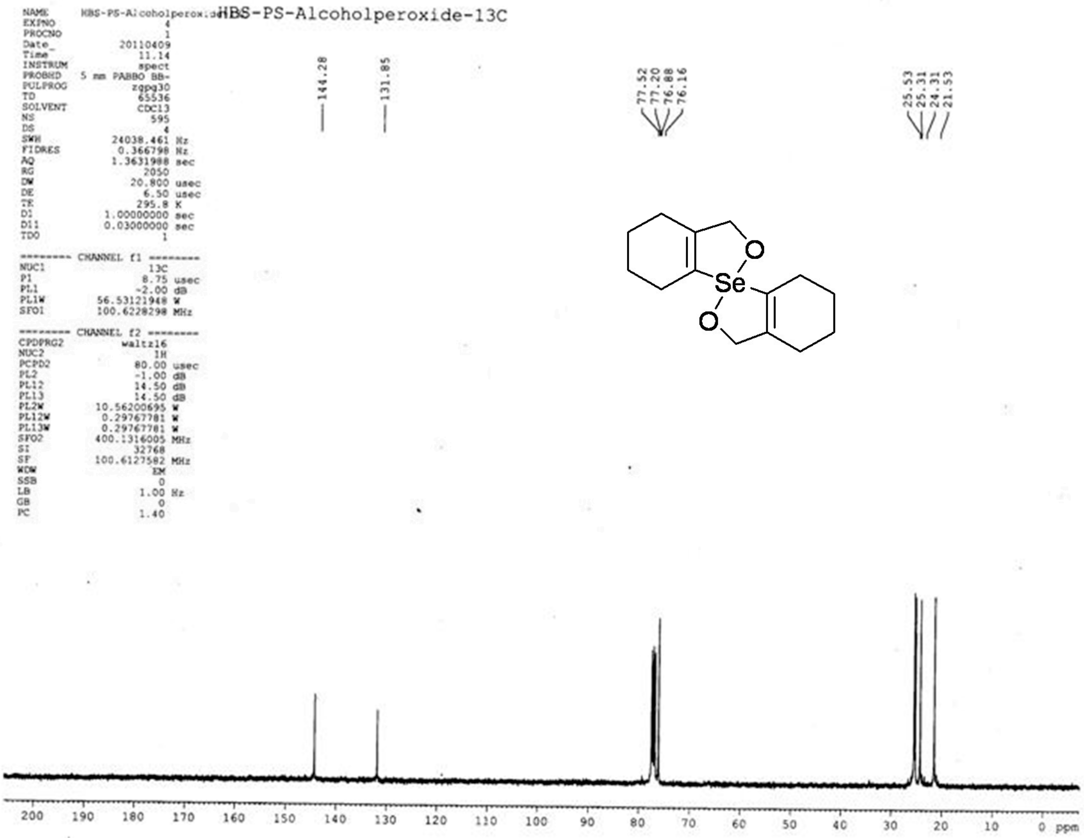

Figure S20. ^{13}C-NMR spectrum of **22** ($CDCl_3$).

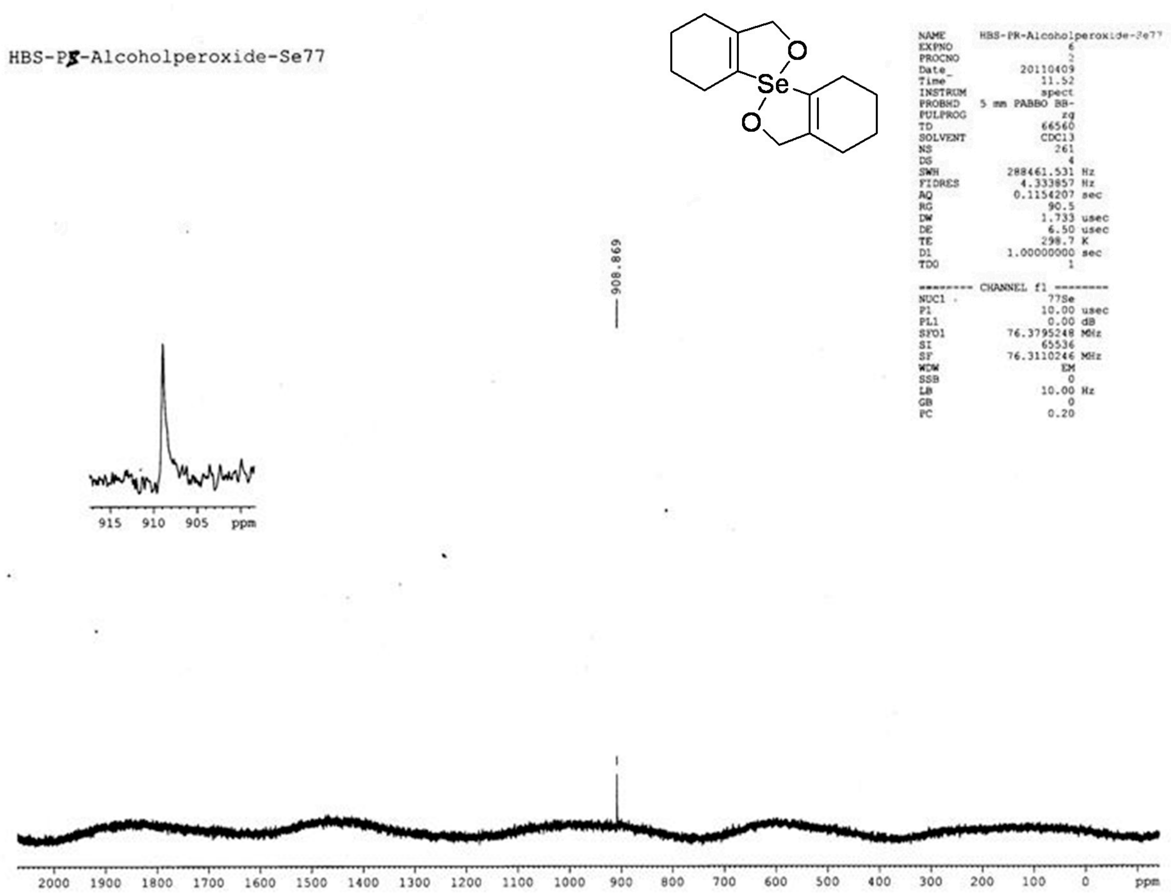

Figure S21. ^{77}Se-NMR spectrum of **22** ($CDCl_3$).

Elemental Composition Report **Page 1**

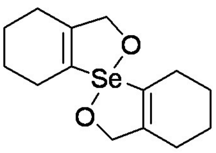

Single Mass Analysis (displaying only valid results)
Tolerance = 10.0 PPM / DBE: min = -1.5, max = 50.0
Isotope cluster parameters: Separation = 1.0 Abundance = 1.0%

Monoisotopic Mass, Odd and Even Electron Ions
49 formula(e) evaluated with 1 results within limits (up to 50 closest results for each mass)

Micromass : Q-Tof micro (YA-105) Dept. Of Chemistry I.I.T.(B) 27-May-201111:49:37

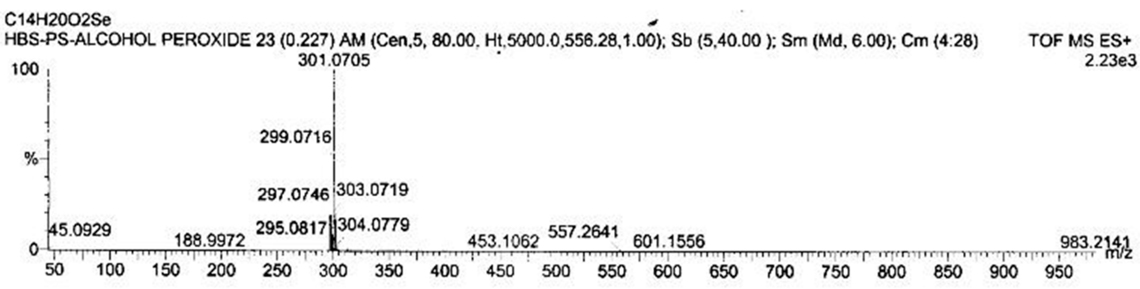

Minimum:				-1.5		
Maximum:		200.0	10.0	50.0		
Mass	Calc. Mass	mDa	PPM	DBE	Score	Formula
301.0705	301.0707	-0.2	-0.7	5.5	1	C14 H21 O2 Se

Figure S22. HRMS spectrum of **22**.

Catalytic mechanisum of di-(2-formylcyclohexenyl)diselenide **16** by ^{77}Se-NMR spectroscopy

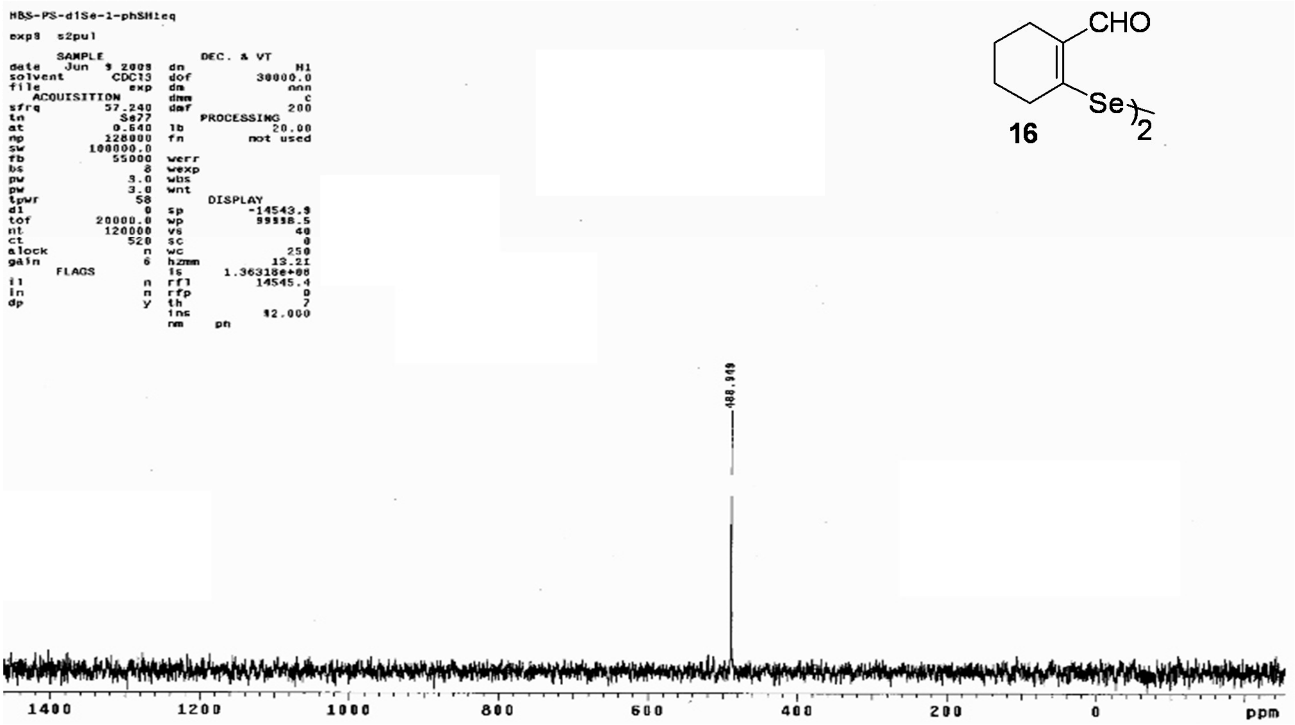

Figure S23. ^{77}Se-NMR spectrum of di-(2-formylcyclohexenyl)diselenide **16** + 1PhSH.

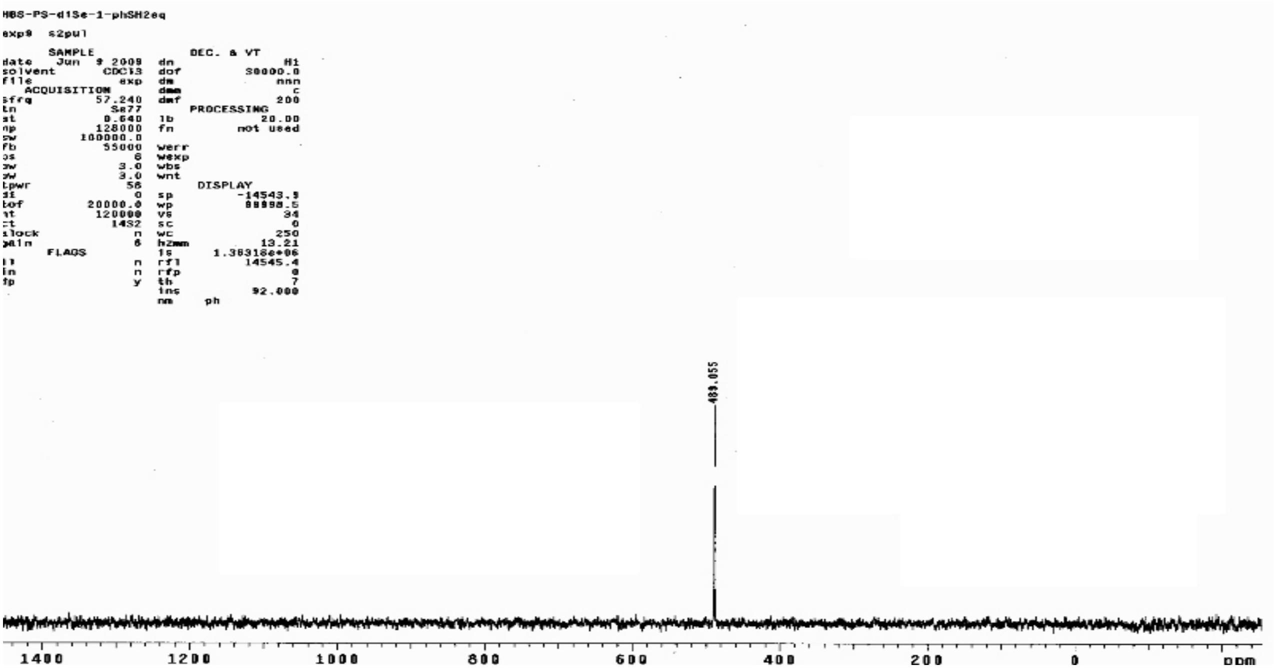

Figure S24. ^{77}Se-NMR spectrum of di-(2-formylcyclohexenyl)diselenide **16** + 2PhSH.

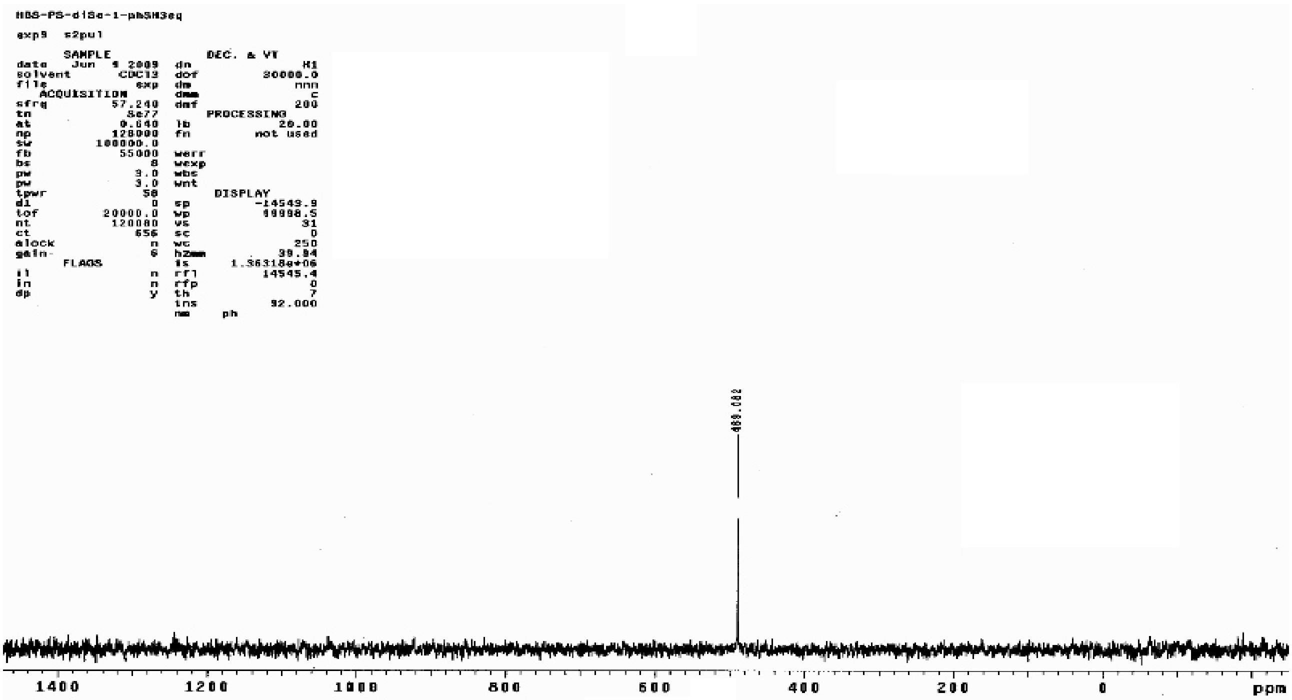

Figure S25. ^{77}Se-NMR spectrum of di-(2-formylcyclohexenyl)diselenide **16** + 3PhSH.

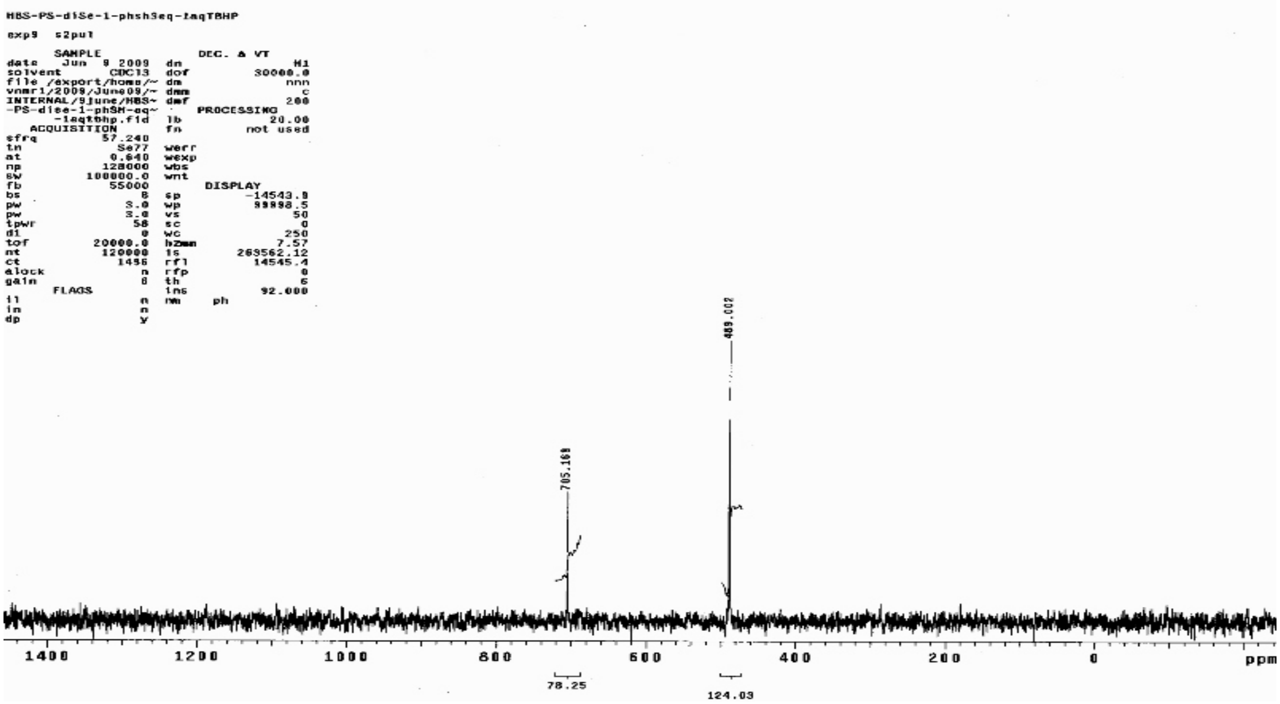

Figure S26. ^{77}Se-NMR spectrum of di-(2-formylcyclohexenyl)diselenide **16** + 2PhSH + 1TBHP.

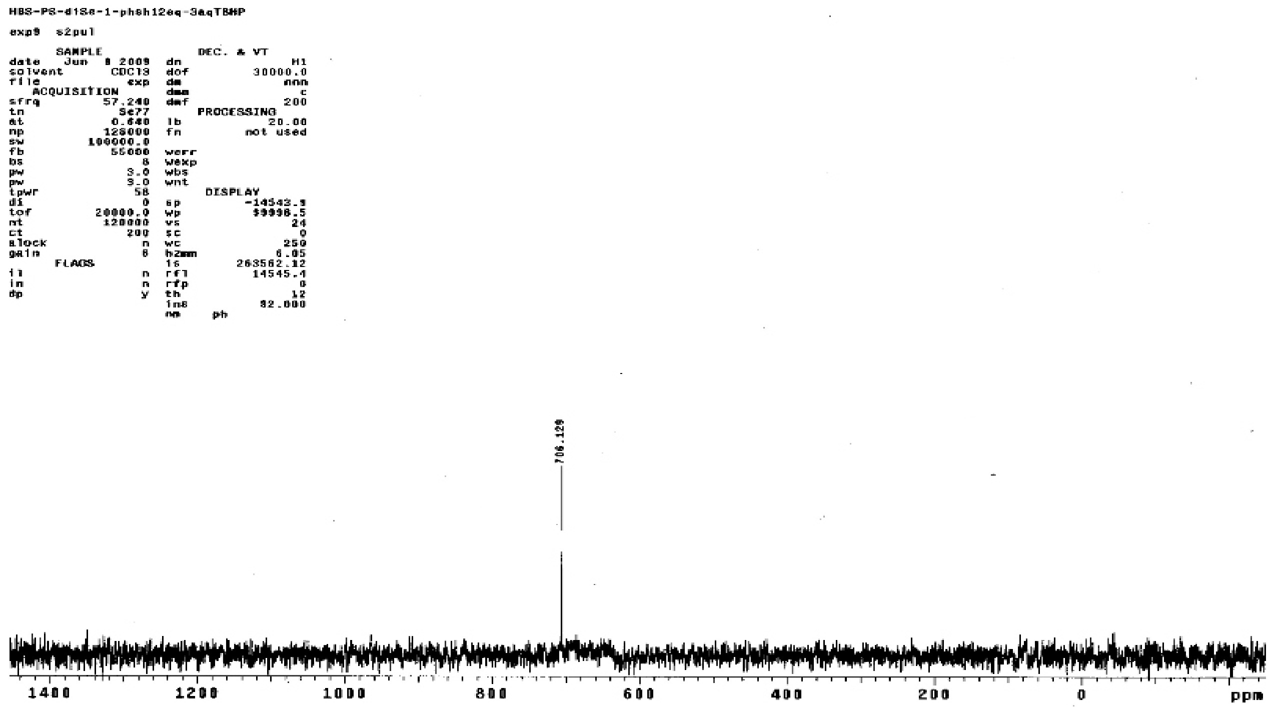

Figure S27. ^{77}Se-NMR spectrum of di-(2-formylcyclohexenyl)diselenide **16** + 2PhSH + 2TBHP.

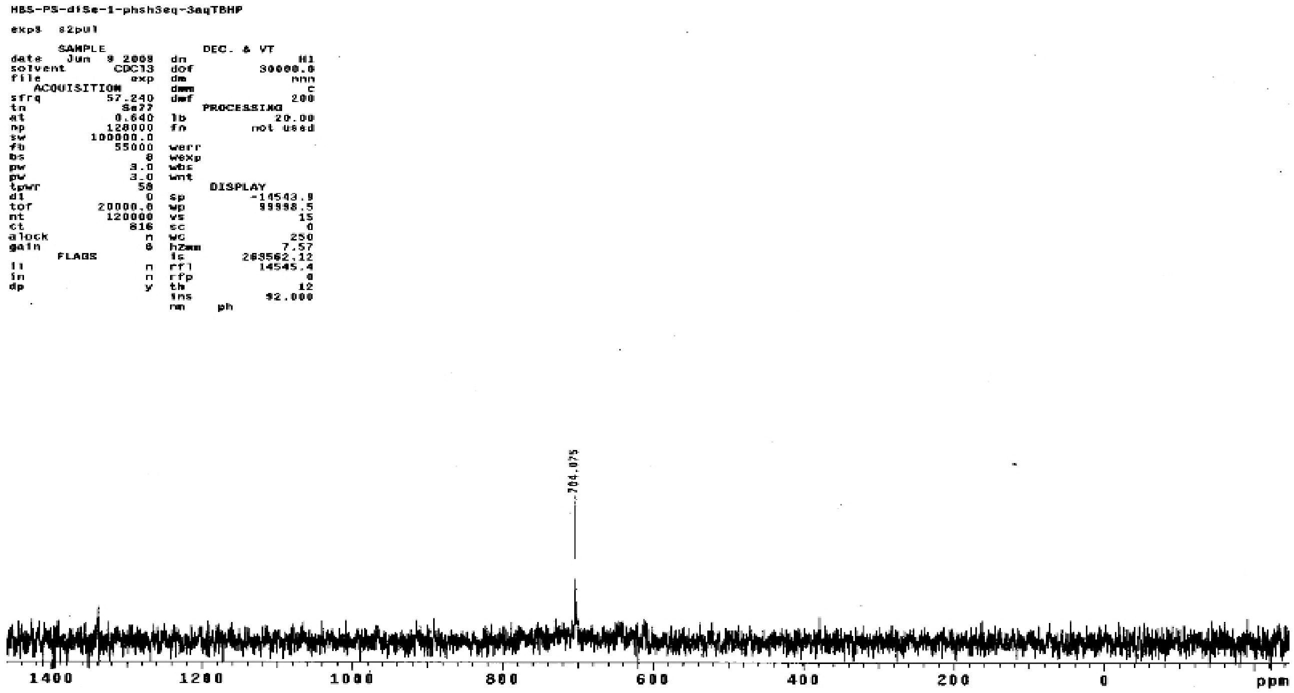

Figure S28. ^{77}Se-NMR spectrum of di-(2-formylcyclohexenyl)diselenide **16** + 2PhSH + 3TBHP.

DFT Calculations and NBO Analysis (in Gas Phase):

Table S1. Optimized geometries of 19 (19a anti-anti; 19b syn-anti) computed at rb3lyp/ 6-31+g(d) and NBO at rb3lyp/6-31+g(d).

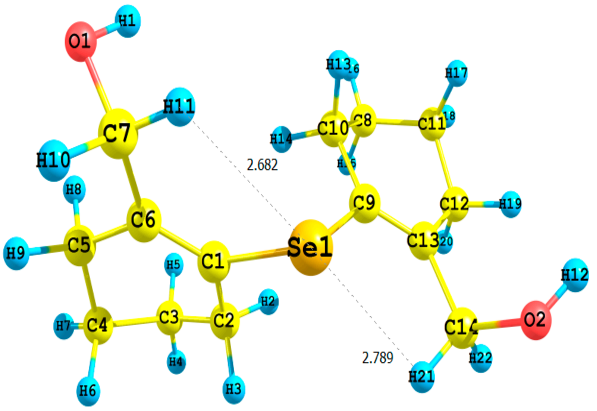

(**19a**)

Electronic Energy = −3096.59172317 a.u.

34	−0.101100000	−1.150920000	−0.550060000
8	3.387008000	0.785287000	−2.475203000
1	2.742266000	1.510171000	−2.450220000
6	1.444640000	−0.330621000	0.312617000
6	1.266934000	−0.094612000	1.796714000
1	0.316572000	0.428109000	1.970251000
1	1.151520000	−1.070446000	2.292775000
6	2.430693000	0.683191000	2.428217000
1	2.368624000	0.615749000	3.521787000
1	2.347338000	1.749280000	2.170629000
6	3.769387000	0.144086000	1.916149000
1	3.853437000	−0.920794000	2.178155000
1	4.609915000	0.656963000	2.400345000
6	3.863165000	0.308115000	0.394486000
1	4.078572000	1.356119000	0.141493000
1	4.716353000	−0.260613000	−0.001424000
6	2.606392000	−0.132396000	−0.344089000
6	2.799521000	−0.355051000	−1.827333000
1	3.519375000	−1.164339000	−1.997770000
1	1.855817000	−0.630488000	−2.308576000
1	−4.393254000	−1.900314000	−0.438502000
1	−1.035408000	1.595676000	−1.753954000
1	−0.047196000	1.851585000	−0.335227000
6	−2.050123000	2.684989000	−0.166292000
1	−1.860810000	2.903418000	0.894752000
1	−1.878959000	3.618234000	−0.717509000
1	−3.683181000	1.990602000	−1.395693000
1	−4.205688000	2.986960000	−0.036840000
8	−3.580891000	−2.321800000	−0.116466000
6	−1.428436000	0.221387000	−0.168544000

6	−1.069576000	1.608061000	−0.653557000
6	−3.495107000	2.205529000	−0.333877000
6	−3.732565000	0.936838000	0.493816000
1	−4.690781000	0.478047000	0.203859000
1	−3.850632000	1.199220000	1.558145000
6	−2.621803000	−0.097251000	0.371290000
6	−2.979378000	−1.477049000	0.879668000
1	−2.089813000	−2.019915000	1.201572000
1	−3.658069000	−1.381152000	1.741764000

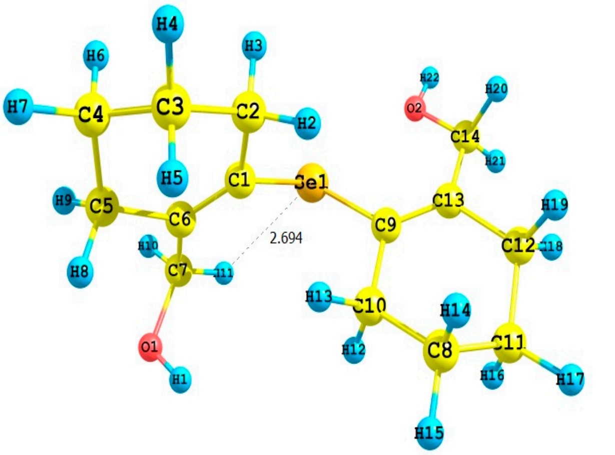

(19b)

Electronic Energy = −3096.58402319 a.u.

34	−0.141084000	−1.157729000	−0.594348000
8	3.195890000	1.101337000	−2.434587000
1	2.516965000	1.782793000	−2.305811000
6	1.399219000	−0.346131000	0.280559000
6	1.261926000	−0.254489000	1.785961000
1	0.303816000	0.221769000	2.035914000
1	1.182628000	−1.275514000	2.188654000
6	2.424125000	0.490559000	2.457965000
1	2.394162000	0.321209000	3.541976000
1	2.310905000	1.573822000	2.302636000
6	3.759733000	0.034268000	1.863618000
1	3.873324000	−1.048264000	2.021917000
1	4.602583000	0.519366000	2.372193000
6	3.806876000	0.341584000	0.361981000
1	3.992248000	1.413908000	0.203975000
1	4.662135000	−0.166299000	−0.106272000
6	2.539763000	−0.058836000	−0.381820000
6	2.694546000	−0.129898000	−1.883856000
1	3.452290000	−0.874920000	−2.153825000
1	1.751023000	−0.411780000	−2.361353000
1	−0.864674000	1.752189000	−1.447885000
1	−0.005287000	1.727395000	0.070109000
6	−1.946287000	2.683580000	0.187753000
1	−1.855454000	2.729125000	1.282945000

1	−1.647177000	3.667183000	−0.196697000
1	−3.488082000	2.329598000	−1.280678000
1	−4.077893000	3.140128000	0.171901000
8	−2.566768000	−2.554805000	0.082843000
6	−1.490564000	0.185206000	−0.145933000
6	−1.004129000	1.607186000	−0.364808000
6	−3.392576000	2.361259000	−0.185787000
6	−3.793738000	1.005370000	0.402603000
1	−4.758386000	0.690142000	−0.021022000
1	−3.970582000	1.114197000	1.485600000
6	−2.764893000	−0.103314000	0.184190000
6	−3.408131000	−1.455632000	0.399141000
1	−3.735752000	−1.514492000	1.452961000
1	−4.323236000	−1.489863000	−0.215895000
1	−3.066882000	−3.375440000	0.211061000

Second Order Perturbation Theory Analysis of Fock Matrix in NBO Basis for 19a

Donor NBO (i) Acceptor NBO (j) kcal/mol a.u. a.u.

**74. LP (2)Se 1**	**/362. BD*(1) C 18 − H 20**	**2.49**	**0.65**	**0.037**
74. LP (2)Se 1	/373. BD*(1) C 30 − C 31	2.06	0.62	0.033
74. LP (2)Se 1	/374. BD*(1) C 30 − C 36	2.12	0.87	0.039
74. LP (2)Se 1	/375. BD*(2) C 30 − C 36	5.82	0.26	0.035
**74. LP (2)Se 1**	**/381. BD*(1) C 37 − H 38**	**1.40**	**0.65**	**0.028**

Coupled Reductase Assay

The GPx-like activities of 16–20 were measured by JASCO spectrophotometer according to the literature method using ebselen as the standard. The catalytic reaction was carried out at room temperature (25 °C) in 1 mL of the solution containing 100 mM potassium phosphate buffer, pH 7.5, pH 7.48, with EDTA (1 mM), NADPH (0.01 M), GSH (0.05 M), catalysts (0.002 M), H_2O_2 (0.026 M), GR (1.3 unit). The activity was followed by the decrease of NADPH on addition of H_2O_2 and absorption was measured at 340 nm (ε_{max} = 6.22 × 10^3 M^{-1} cm^{-1}) (Equations (1)–(3)). Each of the experiments was carried out in triplicate.

$$2GSH + H_2O_2 \xrightarrow[\text{Peroxidase}]{\text{GSH}} GSSG + 2H_2O \qquad (1)$$

$$GSSG + NADPH + H^+ \xrightarrow[\text{Reductase}]{\text{GSH}} 2GSH + NADP^+ \qquad (2)$$

$$NADPH + H_2O_2 + H+ \longrightarrow NADP^+ + 2H20 \qquad (3)$$

For Control	Volume (µL)	For Sample	Volume (µL)
Buffer solution	940	Buffer solution	930
GSH	20	GSH	20
NADPH	25	NADPH	25
GR	5	GR	5
H_2O_2	10	catalyst	10
		H_2O_2	10

Total volume of the solution was taken 1 mL.

Table S2. Glutathione peroxides like activity of compound **control**.

S.N.	ΔA	Initial Reaction Rates (µM min^{-1})
1	0.01790	8.63
2	0.02369	11.43
3	0.02369	11.43

Table S3. Glutathione peroxides like activity of compound **Ebselen**.

S.N.	ΔA	Initial Reaction Rates (µM min^{-1})
1	0.05516	26.60
2	0.05545	26.74
3	0.6249	30.13

Table S4. Glutathione peroxides like activity of compound bis(*o*-formylphenyl)diselenide.

S.N.	ΔA	Initial Reaction Rates (µM min^{-1})
1	0.6664	32.14
2	0.07385	35.61
3	0.07308	32.24

Table S5. Glutathione peroxides like activity of compound **16**.

S.N.	ΔA	Initial Reaction Rates (µM min^{-1})
1	0.0994	47.94
2	0.1056	50.92
3	0.1047	50.49

Table S6. Glutathione peroxides like activity of compound **17**.

S.N.	ΔA	Initial Reaction Rates (µM min^{-1})
1	0.01902	9.17
2	0.02044	9.95
3	0.01863	8.98

Table S7. Glutathione peroxides like activity of compound **19**.

**S.N.**	**ΔA**	**Initial Reaction Rates (μM min^{-1})**
1	0.01300	6.27
2	0.01339	6.45
3	0.01596	7.69

Table S8. Glutathione peroxides like activity of compound **20**.

**S.N.**	**ΔA**	**Initial Reaction Rates (μM min^{-1})**
1	0.02781	13.41
2	0.03607	17.39
3	0.03792	18.28

Table S9. Glutathione peroxides like activity of compound **22**.

**S.N.**	**ΔA**	**Initial Reaction Rates (μM min^{-1})**
1	0.01804	8.70
2	0.01716	8.27
3	0.01802	8.69
