# Supplementary material for: Synthesis, Structure and Antioxidant Activity of Cyclohexene-Fused Selenuranes and Related Derivatives
Source: Molecules. 2015 Jul 13;20(7):12670–85. doi: 10.3390/molecules200712670 (PMC6332442; doi:10.3390/molecules200712670)

checkCIF/PLATON report

No syntax errors found. **CIF dictionary** **Interpreting this report**

Datablock: ps-alse

Bond precision: C-C = 0.0089 A Wavelength=0.71073

**Cell:**	**a=8.7358(12)**	**b=9.1761(12)**	**c=19.689(2)**
	**alpha=90**	**beta=116.096(8)**	**gamma=90**
**Temperature:**	**295 K**		

	Calculated	Reported
Volume	1417.4(3)	1417.4(3)
Space group	P 2/c	P 1 2/c 1
Hall group	-P 2yc	?
Moiety formula	C14 H22 O2 Se	C14 H22 O2 Se
Sum formula	C14 H22 O2 Se	C14 H22 O2 Se
Mr	301.28	301.28
Dx,g cm-3	1.412	1.412
Z	4	4
Mu (mm-1)	2.639	2.639
F000	624.0	624.0
F000’	623.86	
h,k,lmax	10,10,23	10,10,23
Nref	2550	2522
Tmin,Tmax	0.458,0.810	0.251,1.000
Tmin’	0.232	

Correction method= MULTI-SCAN

Data completeness= 0.989 **Theta(max)= 25.190**

R(reflections)= 0.0567(1685) **wR2(reflections)= 0.1529(2522)**

S = 1.024 **Npar= 158**

The following ALERTS were generated. Each ALERT has the format
test-name_ALERT_alert-type_alert-level.
Click on the hyperlinks for more details of the test.

Alert level A

PLAT417_ALERT_2_A Short Inter D-H..H-D H1A .. H1A .. 1.53 Ang.

Alert level C

PLAT241_ALERT_2_C Check High Ueq as Compared to Neighbors for Se
PLAT241_ALERT_2_C Check High Ueq as Compared to Neighbors for C4A

```
PLAT341_ALERT_3_C Low Bond Precision on  C-C Bonds (x 1000) Ang ..          9
PLAT234_ALERT_4_C Large Hirshfeld Difference C3BA   --  C4BA    ..       0.20 Ang.
```

Alert level G

```
PLAT301_ALERT_3_G Note: Main Residue  Disorder ..................      32.00 Perc.
PLAT860_ALERT_3_G Note: Number of Least-Squares Restraints .......         17
PLAT720_ALERT_4_G Number of Unusual/Non-Standard Labels ..........         48
PLAT811_ALERT_5_G No ADDSYM Analysis: Too Many Excluded Atoms ....          !
```

---

1 **ALERT level A** = In general: serious problem
0 **ALERT level B** = Potentially serious problem
4 **ALERT level C** = Check and explain
4 **ALERT level G** = General alerts; check

0 ALERT type 1 CIF construction/syntax error, inconsistent or missing data
3 ALERT type 2 Indicator that the structure model may be wrong or deficient
3 ALERT type 3 Indicator that the structure quality may be low
2 ALERT type 4 Improvement, methodology, query or suggestion
1 ALERT type 5 Informative message, check

Publication of your CIF in IUCr journals

A basic structural check has been run on your CIF. These basic checks will be run on all CIFs submitted for publication in IUCr journals (*Acta Crystallographica, Journal of Applied Crystallography, Journal of Synchrotron Radiation*); however, if you intend to submit to *Acta Crystallographica Section C* or *E*, you should make sure that full publication checks are run on the final version of your CIF prior to submission.

Publication of your CIF in other journals

Please refer to the *Notes for Authors* of the relevant journal for any special instructions relating to CIF submission.

PLATON version of 13/08/2009; check.def file version of 12/08/2009

Datablock ps-alse - ellipsoid plot

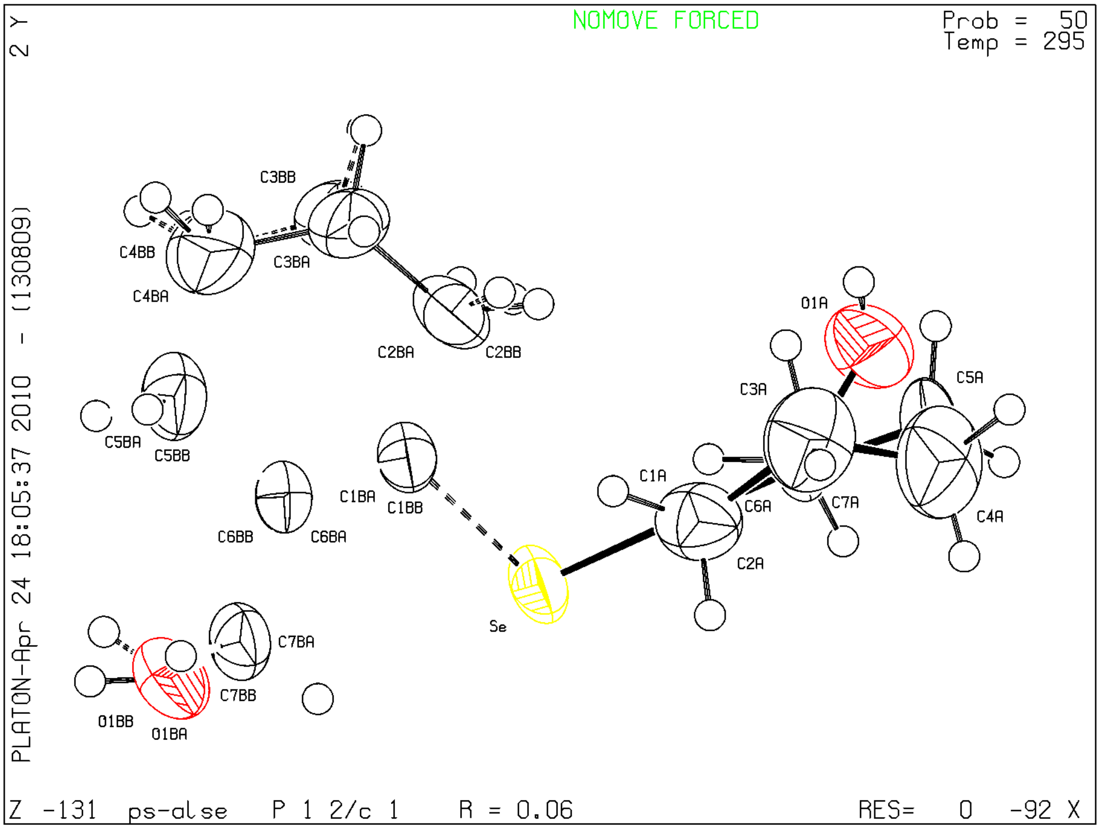

Supplement: Supplementary file 1 [file molecules-20-12670-s001.zip › checkcif/ps-alse_checkcif.pdf]
