# Supplementary material for: Synthesis, Structure and Antioxidant Activity of Cyclohexene-Fused Selenuranes and Related Derivatives
Source: Molecules. 2015 Jul 13;20(7):12670–85. doi: 10.3390/molecules200712670 (PMC6332442; doi:10.3390/molecules200712670)

checkCIF/PLATON report

You have not supplied any structure factors. As a result the full set of tests cannot be run.

THIS REPORT IS FOR GUIDANCE ONLY. IF USED AS PART OF A REVIEW PROCEDURE FOR PUBLICATION, IT SHOULD NOT REPLACE THE EXPERTISE OF AN EXPERIENCED CRYSTALLOGRAPHIC REFEREE.

No syntax errors found. CIF dictionary Interpreting this report

Datablock: ups817

```
Bond precision:  C-C = 0.0020 A          Wavelength=0.71073

Cell:           a=15.0340(13)    b=8.5925(7)        c=10.4102(8)
                alpha=90         beta=112.358(4)    gamma=90
Temperature:    100 K

                Calculated                Reported
Volume          1243.69(18)               1243.69(18)
Space group     C 2/c                     C 2/c
Hall group      -C 2yc                    ?
Moiety formula  C14 H16 O4 Se             C14 H16 O4 Se
Sum formula     C14 H16 O4 Se             C56 H64 O16 Se4
Mr              327.23                    1308.91
Dx,g cm-3       1.748                     1.748
Z               4                         1
Mu (mm-1)       3.027                     3.027
F000            664.0                     664.0
F000'           663.94
h,k,lmax        21,12,15                  21,12,14
Nref            1972                      1963
Tmin,Tmax       0.390,0.514               0.542,0.746
Tmin'           0.333

Correction method= NONE

Data completeness= 0.995          Theta(max)= 30.910

R(reflections)= 0.0237( 1830)     wR2(reflections)= 0.0609( 1963)

S = 1.099                   Npar= 87
```

The following ALERTS were generated. Each ALERT has the format
test-name_ALERT_alert-type_alert-level.
Click on the hyperlinks for more details of the test.

Alert level A

```
ABSTY03_ALERT_1_A  The _exptl_absorpt_correction_type has been given as none.
            However a value has been given for _exptl_absorpt_process_details.
  From the CIF: _exptl_absorpt_process_details         Sadabs (Sheldrick, 1996)
PLAT057_ALERT_3_A Correction for Absorption Required   RT(exp) ...       1.32 Do !
```

Alert level C

```
ABSTY03_ALERT_1_C  The _exptl_absorpt_correction_type has been given as none.
            However values have been given for Tmin and Tmax. Remove
            these if an absorption correction has not been applied.
  From the CIF: _exptl_absorpt_correction_T_min   0.542
  From the CIF: _exptl_absorpt_correction_T_max   0.746
PLAT094_ALERT_2_C Ratio of Maximum / Minimum Residual Density ....       2.18 Report
PLAT480_ALERT_4_C Long H...A H-Bond Reported H3     ..  O2A     ..       2.61 Ang.
```

Alert level G

```
FORMU01_ALERT_1_G  There is a discrepancy between the atom counts in the
            _chemical_formula_sum and _chemical_formula_moiety. This is
            usually due to the moiety formula being in the wrong format.
            Atom count from _chemical_formula_sum:   C56 H64 O16 Se4
            Atom count from _chemical_formula_moiety:C14 H16 O4 Se1
PLAT005_ALERT_5_G No _iucr_refine_instructions_details  in the CIF     Please Do !
PLAT045_ALERT_1_G Calculated and Reported Z Differ by ...........       4.00 Ratio
PLAT128_ALERT_4_G Alternate Setting for Input Space Group     C2/c       I2/a Note
PLAT710_ALERT_4_G Delete 1-2-3 or 2-3-4 Linear Torsion Angle ... #          3 Do !
            O1A -SE  -O1A -C7A    56.79  0.10   2.656   1.555   1.555   1.555
PLAT899_ALERT_4_G SHELXL97   is Deprecated and Succeeded by SHELXL       2014 Note
```

---

2 **ALERT level A** = Most likely a serious problem - resolve or explain
0 **ALERT level B** = A potentially serious problem, consider carefully
3 **ALERT level C** = Check. Ensure it is not caused by an omission or oversight
6 **ALERT level G** = General information/check it is not something unexpected

4 ALERT type 1 CIF construction/syntax error, inconsistent or missing data
1 ALERT type 2 Indicator that the structure model may be wrong or deficient
1 ALERT type 3 Indicator that the structure quality may be low
4 ALERT type 4 Improvement, methodology, query or suggestion
1 ALERT type 5 Informative message, check

It is advisable to attempt to resolve as many as possible of the alerts in all categories. Often the minor alerts point to easily fixed oversights, errors and omissions in your CIF or refinement strategy, so attention to these fine details can be worthwhile. In order to resolve some of the more serious problems it may be necessary to carry out additional measurements or structure refinements. However, the purpose of your study may justify the reported deviations and the more serious of these should normally be commented upon in the discussion or experimental section of a paper or in the "special_details" fields of the CIF. checkCIF was carefully designed to identify outliers and unusual parameters, but every test has its limitations and alerts that are not important in a particular case may appear. Conversely, the absence of alerts does not guarantee there are no aspects of the results needing attention. It is up to the individual to critically assess their own results and, if necessary, seek expert advice.

Publication of your CIF in IUCr journals

A basic structural check has been run on your CIF. These basic checks will be run on all CIFs submitted for publication in IUCr journals (*Acta Crystallographica*, *Journal of Applied Crystallography*, *Journal of Synchrotron Radiation*); however, if you intend to submit to *Acta Crystallographica Section C* or *E*, you should make sure that full publication checks are run on the final version of your CIF prior to submission.

Publication of your CIF in other journals

Please refer to the *Notes for Authors* of the relevant journal for any special instructions relating to CIF submission.

Validation response form

Please find below a validation response form (VRF) that can be filled in and pasted into your CIF.

```
# start Validation Reply Form
_vrf_ABSTY03_ups817
;
PROBLEM: The _exptl_absorpt_correction_type has been given as none.
RESPONSE: ...
;
_vrf_PLAT057_ups817
;
PROBLEM: Correction for Absorption Required   RT(exp) ...       1.32 Do !
RESPONSE: ...
;
_vrf_PLAT094_ups817
;
PROBLEM: Ratio of Maximum / Minimum Residual Density ....       2.18 Report
RESPONSE: ...
;
_vrf_PLAT480_ups817
;
PROBLEM: Long H...A H-Bond Reported H3     ..  O2A     ..       2.61 Ang.
RESPONSE: ...
;
# end Validation Reply Form
```

PLATON version of 20/08/2014; check.def file version of 18/08/2014

Datablock ups817 - ellipsoid plot

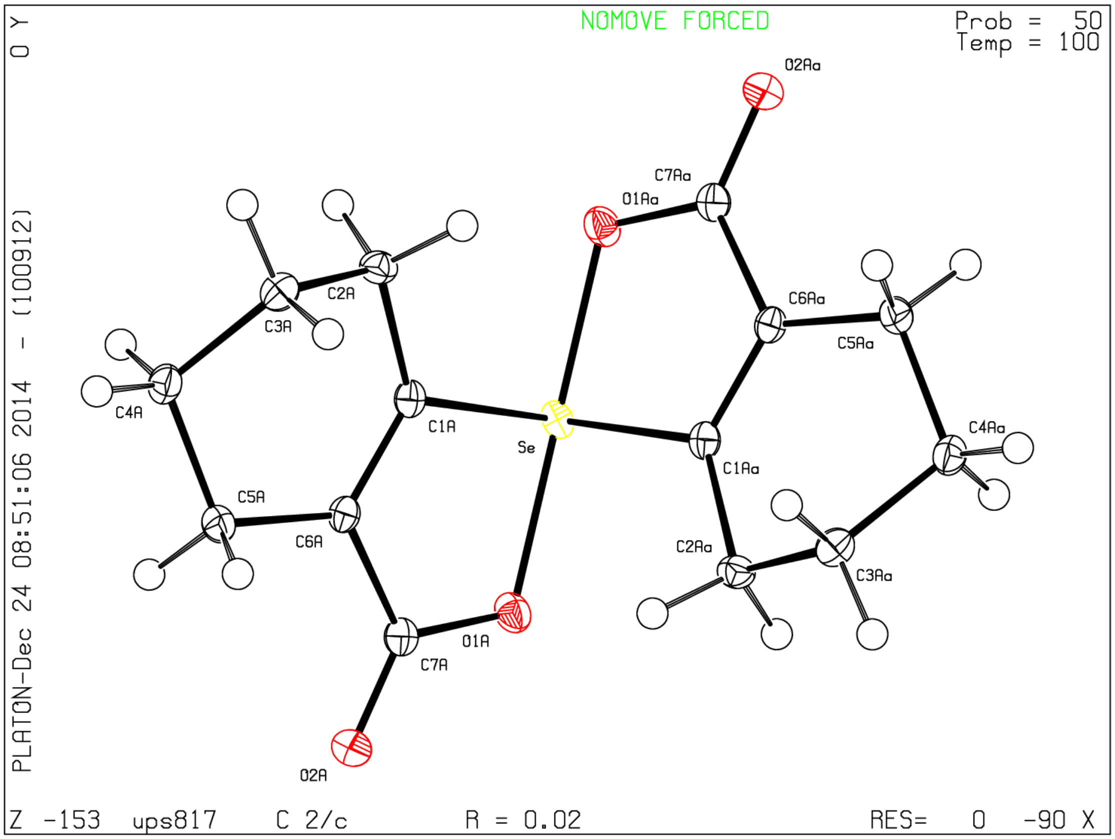

Supplement: Supplementary file 1 [file molecules-20-12670-s001.zip › checkcif/sespirocheckcif.pdf]
