# Supplementary material for: Synthesis, Structure and Antioxidant Activity of Cyclohexene-Fused Selenuranes and Related Derivatives
Source: Molecules. 2015 Jul 13;20(7):12670–85. doi: 10.3390/molecules200712670 (PMC6332442; doi:10.3390/molecules200712670)

checkCIF/PLATON report

No syntax errors found. **CIF dictionary** **Interpreting this report**

Datablock: te-spiro

Bond precision: O- C = 0.0052 A Wavelength=1.54184

**Cell:**	**a=15.7046(2)**	**b=8.83038(16)**	**c=19.1603(3)**
	**alpha=90**	**beta=90**	**gamma=90**
**Temperature:**	**123 K**		

	Calculated	Reported
Volume	2657.11(7)	2657.11(8)
Space group	P b c a	P b c a
Hall group	-P 2ac 2ab	?
Moiety formula	C14 H16 O4 Te	C14 H16 O4 Te
Sum formula	C14 H16 O4 Te	C14 H16 O4 Te
Mr	375.87	375.87
Dx,g cm-3	1.879	1.879
Z	8	8
Mu (mm-1)	17.759	17.759
F000	1472.0	1472.0
F000’	1475.02	
h,k,lmax	19,10,23	19,10,23
Nref	2676	2443
Tmin,Tmax	0.014,0.142	0.233,1.000
Tmin’	0.002	

Correction method= MULTI-SCAN

Data completeness= 0.913 Theta(max)= 73.540

R(reflections)= 0.0480(2127) wR2(reflections)= 0.1330(2443)

S = 1.039 Npar= 210

The following ALERTS were generated. Each ALERT has the format
test-name_ALERT_alert-type_alert-level.
Click on the hyperlinks for more details of the test.

Alert level A

PLAT029_ALERT_3_A _diffrn_measured_fraction_theta_full Low 0.93

Alert level C

PLAT022_ALERT_3_C Ratio Unique / Expected Reflections too Low 0.91

Alert level G

```
PLAT301_ALERT_3_G Note: Main Residue  Disorder ..................      39.00 Perc.
PLAT432_ALERT_2_G Short Inter X...Y Contact  Te     ..  O2A     ..       2.94 Ang.
PLAT432_ALERT_2_G Short Inter X...Y Contact  Te     ..  O2B     ..       3.33 Ang.
PLAT860_ALERT_3_G Note: Number of Least-Squares Restraints .......         72
PLAT720_ALERT_4_G Number of Unusual/Non-Standard Labels ..........         56
PLAT779_ALERT_4_G Suspect or Irrelevant (Bond) Angle in CIF .... #          7
              C1AA -TE   -C1AB    1.555   1.555   1.555              1.30 Deg.
PLAT779_ALERT_4_G Suspect or Irrelevant (Bond) Angle in CIF .... #         13
              C1BB -TE   -C1BA    1.555   1.555   1.555              2.20 Deg.
PLAT779_ALERT_4_G Suspect or Irrelevant (Bond) Angle in CIF .... #         82
              C6AB -C7A  -C6AA    1.555   1.555   1.555              2.30 Deg.
PLAT779_ALERT_4_G Suspect or Irrelevant (Bond) Angle in CIF .... #        149
              C6BB -C7B  -C6BA    1.555   1.555   1.555              2.80 Deg.
PLAT811_ALERT_5_G No ADDSYM Analysis: Too Many Excluded Atoms ....          !
```

---

1 **ALERT level A** = In general: serious problem
0 **ALERT level B** = Potentially serious problem
1 **ALERT level C** = Check and explain
10 **ALERT level G** = General alerts; check

0 ALERT type 1 CIF construction/syntax error, inconsistent or missing data
2 ALERT type 2 Indicator that the structure model may be wrong or deficient
4 ALERT type 3 Indicator that the structure quality may be low
5 ALERT type 4 Improvement, methodology, query or suggestion
1 ALERT type 5 Informative message, check

Publication of your CIF in IUCr journals

A basic structural check has been run on your CIF. These basic checks will be run on all CIFs submitted for publication in IUCr journals (*Acta Crystallographica*, *Journal of Applied Crystallography*, *Journal of Synchrotron Radiation*); however, if you intend to submit to *Acta Crystallographica Section C* or *E*, you should make sure that full publication checks are run on the final version of your CIF prior to submission.

Publication of your CIF in other journals

Please refer to the *Notes for Authors* of the relevant journal for any special instructions relating to CIF submission.

PLATON version of 22/10/2010; check.def file version of 11/10/2010

Datablock te-spiro - ellipsoid plot

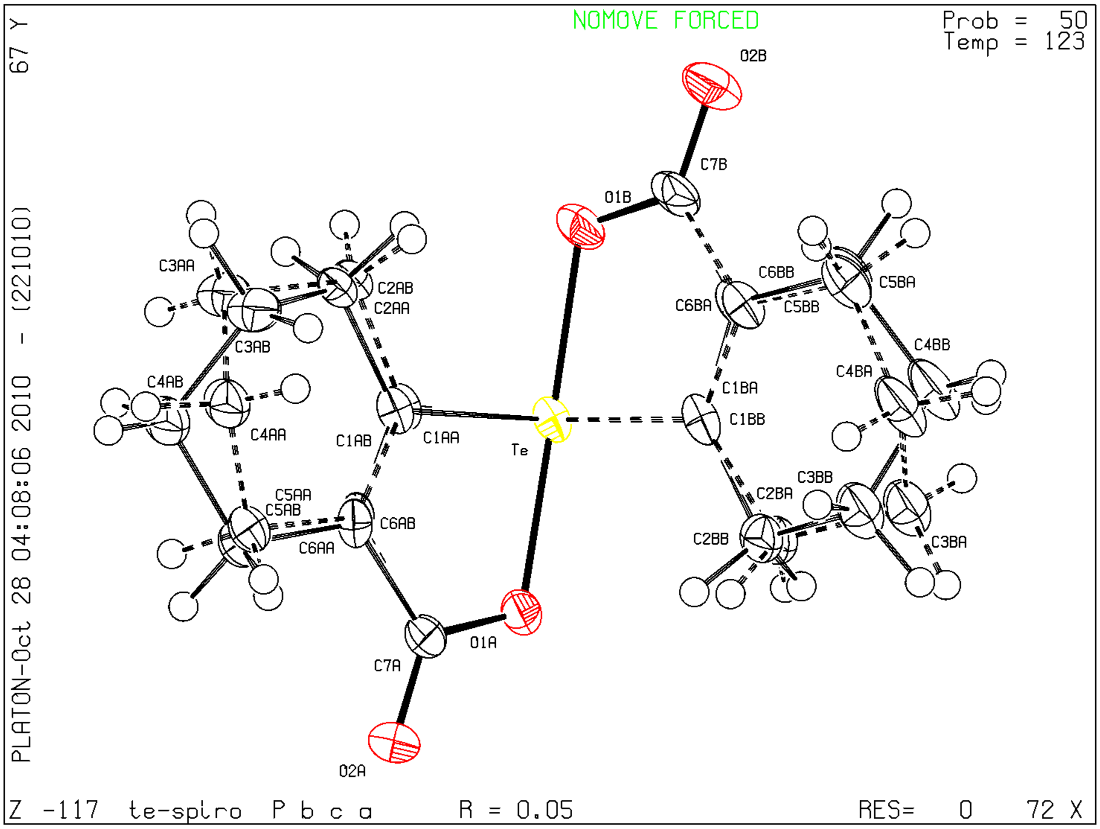

Supplement: Supplementary file 1 [file molecules-20-12670-s001.zip › checkcif/te-spiro_checkcif.pdf]
